# Discovery of Novel Dual Extracellular Regulated Protein Kinases (ERK) and Phosphoinositide 3-Kinase (PI3K) Inhibitors as a Promising Strategy for Cancer Therapy

**DOI:** 10.3390/molecules25235693

**Published:** 2020-12-03

**Authors:** Lingzhi Zhang, Qiurong Ju, Jinjin Sun, Lei Huang, Shiqi Wu, Shuping Wang, Yin Li, Zhe Guan, Qihua Zhu, Yungen Xu

**Affiliations:** 1 State Key Laboratory of Natural Medicines, China Pharmaceutical University, Nanjing 210009, China; zhanglingzhicpu@163.com (L.Z.); jqr474678912@163.com (Q.J.); fas15850601108@163.com (J.S.); 18361073522@163.com (L.H.); wushiqicpu1@163.com (S.W.); wangsp16@126.com (S.W.); linny_cpu@126.com (Y.L.); guanz2008@163.com (Z.G.); 2 Jiangsu Key Laboratory of Drug Design and Optimization, Department of Medicinal Chemistry, China Pharmaceutical University, Nanjing 210009, China

**Keywords:** ERK inhibitor, PI3K inhibitor, dual ERK/PI3K inhibitors, cross-talk

## Abstract

Concomitant inhibition of MAPK and PI3K signaling pathways has been recognized as a promising strategy for cancer therapy, which effectively overcomes the drug resistance of MAPK signaling pathway-related inhibitors. Herein, we report the scaffold-hopping generation of a series of 1*H*-pyrazolo[3,4-*d*]pyrimidine dual ERK/PI3K inhibitors. Compound **32d** was the most promising candidate, with potent inhibitory activities against both ERK2 and PI3Kα which displays superior anti-proliferative profiles against HCT116 and HEC1B cancer cells. Meanwhile, compound **32d** possessed acceptable pharmacokinetic profiles and showed more efficacious anti-tumor activity than GDDC-0980 and the corresponding drug combination (BVD-523 + GDDC-0980) in HCT-116 xenograft model, with a tumor growth inhibitory rate of 51% without causing observable toxic effects. All the results indicated that **32d** was a highly effective anticancer compound and provided a promising basis for further optimization towards dual ERK/PI3K inhibitors.

## 1. Introduction

Numerous small molecule kinase inhibitors have become an effective methods for the treatment of cancer, due to their good selectivity, high potency and low toxicity. However, because of drug resistance, the clinical benefits of small molecule kinase inhibitors have been greatly limited [1]. The resistance mechanism can be divided into two categories: one is from the overexpression and resistance mutations of the target kinase itself (on-target); the other is not directly related to the target itself, but through alternative signaling pathways to achieve resistance (by-pass) [2]. Therefore, in addition to the continuous development of new kinase inhibitors targeting mutation sites, the drug resistance could be also better overcome by inhibiting related bypass signaling pathways. Now, drug combination strategy is performed to overcome drug resistance by synergy effects of two or more drugs. However, there are some drawbacks of drug combination, such as incompatible PK, enhanced toxicity, or even causing drug-induced diseases and threatening life. On the contrary, multi-target drugs not only simultaneously act on multiple targets to exert a synergistic effect, but also reduce the dosage of drugs, avoiding the safety problems caused by the interaction between drugs. In addition, uniform pharmacokinetic properties are beneficial to precise drug therapy in the human body. In 2019, a clinical trial of the combination therapy of MEK Inhibitor Binimetinib and phosphatidylinositol 3-kinase (PI3K) inhibitor Buparlisib was terminated in patients with advanced solid tumors with RAS/RAF alterations because of adverse events [3]. Therefore, the development of dual MAPK and PI3K pathways inhibitors has great potential application prospects.

**Figure 1:**
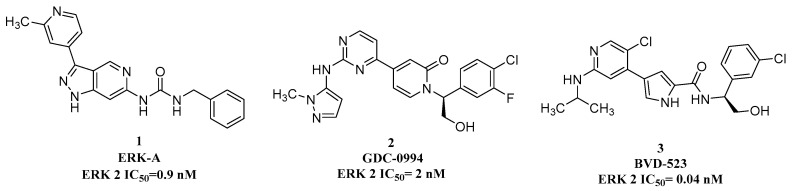
Structures of representative ERK inhibitors.

The mitogen-activated protein kinases (MAPK) pathway, often known as the RAS-RAF-MEK-ERK signal cascade and activated through polypeptide or growth factors that bind to transmembrane receptor tyrosine kinases (RTKs), exhibits the ability to transmit upstream signals to its downstream effectors to regulate physiological processions such as cell proliferation, differentiation, survival and death. As the most frequently mutated signaling pathway in human cancer, targeting the MAPK pathway has long been considered as a promising strategy for cancer therapy. Now several drugs targeting this pathway have been approved by the FDA, such as BRAF inhibitors (Vemurafenib and Dabrafenib [4,5]) and MEK inhibitors (Trametinib, Cobimetinib and Selumetinib [6,7]). Despite the considerable success of BRAF and MEK inhibitors, with clinical studies carried out, most patients experience recurrence in less than a year and about 10~15% of patients harboring B-RafV600E are insensitive to BRAF and MEK inhibitors due to the amplification and mutation of BRAF/MEK and the negative feedback loops [8], resulting in the limiting of further clinical application. In such cases, targeting ERK, a downstream key node of BRAF/MEK, has been proposed as a potential strategy for overcoming acquired drug resistance. Preclinical studies suggest that ERK inhibitors have an advantage over inhibiting the growth of BRAF/RAS mutated tumor and overcoming BRAF or/and MEK inhibitor resistance. Representative drugs have ERK-A (**1**) [9], GDC-0994 (**2**) [10], BVD-523 (**3**), etc. (Figure 1).

**Figure 2:**
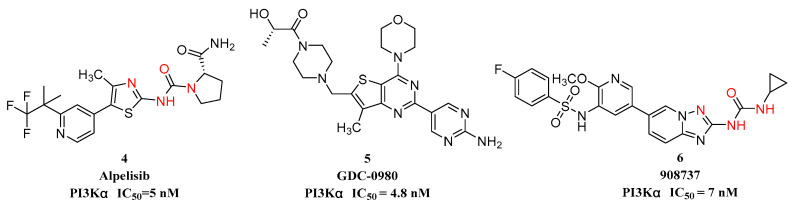
Structures of representative PI3K inhibitors.

Phosphoinositide 3-kindase (PI3K) is a conserved family of lipid kinases, which is composed of the catalytic subunit p110 and the regulatory subunit p85 or p101. PI3K can phosphorylate the 3-hydroxyl of phosphatidylinositol 4,5-bisphosphate (PIP2) to generate phosphatidylinositol 3,4,5-trisphosphate, PIP3) [11]. As a second messenger, PIP3 plays an important role in cell survival, growth, proliferation and metabolism. The tumor suppressor gene PTEN (phosphatase and tension homolog deleted on chromosome ten) can dephosphorylate PIP3 to generate PIP2, which is an antagonist of the catalytic effect of PI3K [12]. However, it was found that PTEN was the most mutated tumor suppressor gene [13] in endometrial tumors, central nervous system diseases, skin cancer and prostate cancer. Cancer gene research has shown that the PI3K signaling pathway is the most susceptible to mutation in human tumors, and has been a popular target for tumor treatment. Representative drugs have Alpelisib (**4**) [14], GDC-0980 (**5**) [15], 908737 (**6**) [16], etc. (Figure 2)

**Figure 3:**
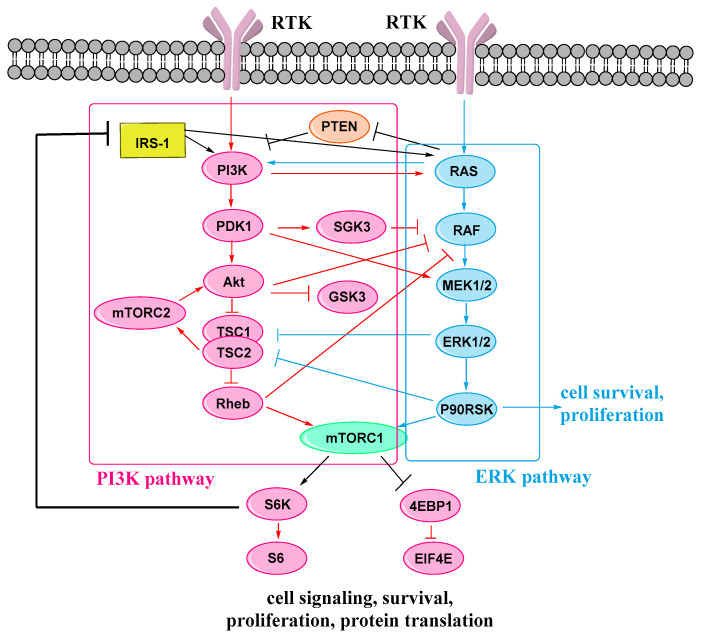
The cross-talk between MAPK/ERK and PI3K/Akt pathways.

Studies have found that there are multiple feedback adjustments between ERK signaling pathway and PI3K signaling pathway, and combined blockage of ERK and PI3K pathways significantly decreased cell viability, compared with single target inhibition [17]. The interaction between the PI3K and ERK pathways is very complex and has not yet been fully understood [14]. Firstly, in both physiological and pathological conditions, there is a significant level of cross-talk between kinases of these two pathways (Figure 3). Phosphorylated PI3K could activate RAS via several mechanisms [18], thereby reactivating the ERK pathway. Meanwhile, inhibition of PDK1, AKT or Rheb could increase RAF activities [19,20,21]. Besides, the classic feedback loop (S6-IRS1-PI3K) leads to activation of PI3K but also ERK signaling [22]. PI3K and ERK cascade signaling converge on mTORC1, which is the master regulator of ribosome biogenesis and protein translation. To sum up, when one pathway is inhibited, the other would be activated by cross-talk or feedback loops, thereby promoting the development of tumors. Therefore, the design of ERK and PI3K dual inhibitors is expected to better overcome the drug resistance of ERK pathway-related inhibitors and obtain better clinical benefits. So far, no dual ERK/PI3K inhibitors are available in the clinic or the market. In this work, we designed a series of compounds derived from ERK-A as ERK and PI3K dual inhibitors. Further optimization culminated in the identification of a potent compound **32d** exhibiting the best inhibitory activity against both ERK and PI3K enzymes.

## 2. Results and Discussion

### 2.1. Drug Design

**Figure 4:**
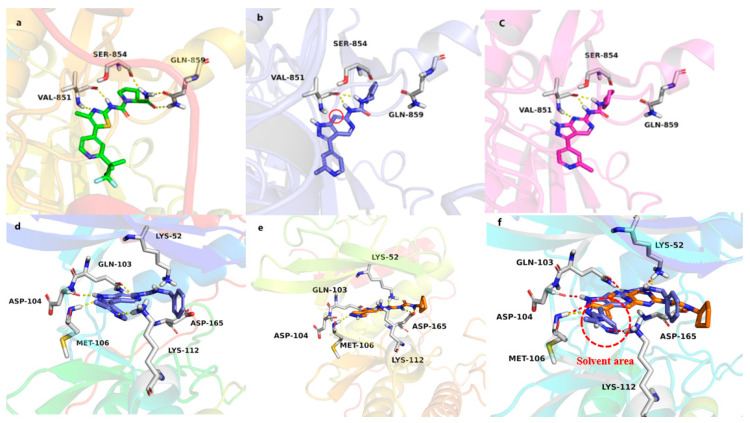
The 3D interactions diagram of complex structures (**a**~**c**: PDB code 4JPS; **d**~**f**: PDB code 5KE0). (**a**) X-ray structure of Alpelisib (green) bound to PI3Kα; (**b**) ERK-A (purple) docked into X-ray structure of PI3Kα. (**c**) **16b** (red) docked into X-ray structure of PI3Kα. (**d**) X-ray structure of ERK-A (purple) bound to ERK 2; (**e**) **32d** (orange) docked into X-ray structure of ERK 2; (**f**) Overlap of the proposed binding modes of ERK-A (purple) and **32d** (orange) in ERK2. The figure was generated using PyMol (http://www.pymol.org/).

**Figure 5:**
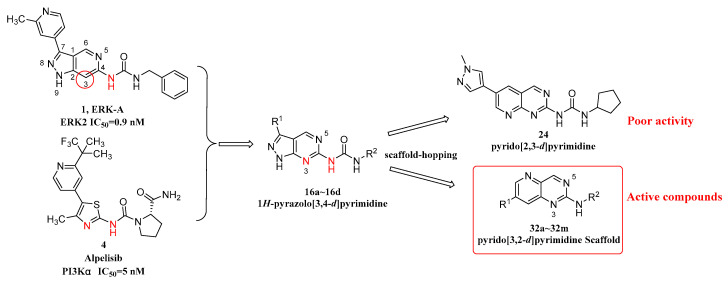
Design of our ERK/PI3K dual from 1*H*-pyrazolo[4,3-*c*]pyridine to pyrido[3,2-*d*]pyrimidine scaffold.

In the process of investigating existing PI3K inhibitors, we found that a urea-based with Nitrogen-containing heterocycle was often introduced into PI3K inhibitors, such as compound **4** and compound 908737 (Figure 2, the structure marked in red). Meanwhile, in the structure of an ATP competitive ERK inhibitor ERK-A [6], it also was found to contain a structure similar to the urea-based with heterocycle. Therefore, we docked the compound ERK-A into PI3Kα protein (PDB ID: 4JPS) (Figure 4b) and compared the co-crystal structure of Alpelisib (**4**, PI3Kα selective inhibitor) (Figure 4a). It was found that when a N atom was introduced at the C3 position, it could form two hydrogen bonding interactions with the Val851 in the PI3Kα protein skeleton region which is vital for maintaining the PI3Kα inhibition activities (Figure 4b,c). Thus, we designed and synthesized the 1*H*-pyrazolo[3–*d*]pyrimidine derivatives **16a**~**16d**, as shown in Figure 5. After preliminary kinase inhibitory activity studies, to our surprise, compound **16b** exhibited 27.4% PI3Kα inhibitory activity at 1 μM while retaining good ERK2 inhibitory activity. To further improve the PI3K inhibitory activity, we replaced the 1*H*-pyrazolo[3,4-*d*]pyrimidine scaffold with the pyrido[3,2-*d*]pyrimidine or pyrido[2,3-*d*]pyrimidine through scaffold-hopping strategy to synthesize **24** and **32a**~**32m** (Figure 5). After docking **32d** with the best potency to the co-crystal structure of ERK2 (PDB ID: 5KE0), it was found that N1 of compound **32d** could form a hydrogen bonding interaction with the Lys52 in the ERK2 protein skeleton region which is vital for maintaining the ERK2 inhibition activities (Figure 4e); meanwhile, the pyrazole substituent of **32d** extended into the solvent accessible area (Figure 4f).

### 2.2. Chemistry

**Scheme 1:**
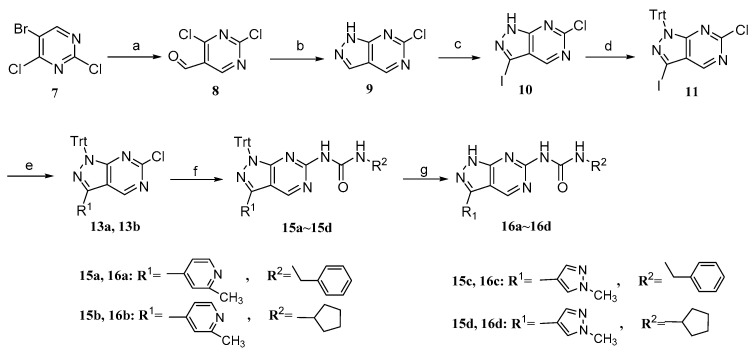
Preparation of target compounds **16a**~**16d**. Reagents and conditions: (**a**) 1.3 M i-PrMgCl**·**LiCl, dry DMF, THF, −78~−42 °C, 24 h; (**b**) 80% N_2_H_4_**·**H_2_O, THF, 0 °C, 1 h; (**c**) NIS, DMF, rt, 12 h; (**d**) TrtCl, K_2_CO_3_, MeCN, rt, 12 h; (**e**) (2-methyl pyridin-4-yl)boronic acid (**12a**) or 1-methyl-4-(4,4,5,5-tetramethyl-1,3,2-dioxaborolan-2-yl)-1*H*-pyrazole (**12b**), Pd(dppf)Cl_2_, K_2_CO_3_, V_(1,4-dioxane)_:V_(H2O)_ = 5:1, N_2_, 60 °C, 2 h; (**f**) Cyclopentylurea (**14a**) or Benzyl urea (**14b**), Pd_2_(dba)_3_, Xantphos, Cs_2_CO_3_, PhMe, N_2_, 110 °C, 3 h; (**g**) F_3_CCO_2_H, DCM, rt, 12 h.

**Scheme 2:**
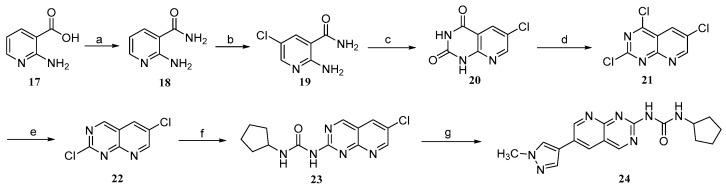
Preparation of target compounds **24**. Reagents and conditions: (**a**) NH_4_Cl, HOBt, DIEA, EDCI, DMF, rt, 12 h; (**b**) 30% H_2_O_2_, conc. HCl, 60 °C, 6 h; (**c**) Diphosgene, 120 °C,12 h; (**d**) POCl_3_, DMF, 130 °C; (**e**) Bu_3_SnH, Pd(PPh_3_)_4_, PhMe, N_2_, 100 °C,1 h; (**f**) Cyclopentylurea (**14a**), Pd(OAc)_2_, XantPhos, t-BuOK, 1,4-dioxane, 60 °C, N_2_, 2 h. (**g**) 1-methyl-4-(4,4,5,5-tetramethyl-1,3,2-dioxa borolan-2-yl)-1*H*-pyrazole (**12b**), Pd(dppf)Cl_2_, K_2_CO_3_, V_(1,4-dioxane)_:V_(H2O)_ = 5:1, 60 °C, N_2_, 2 h.

**Scheme 3:**
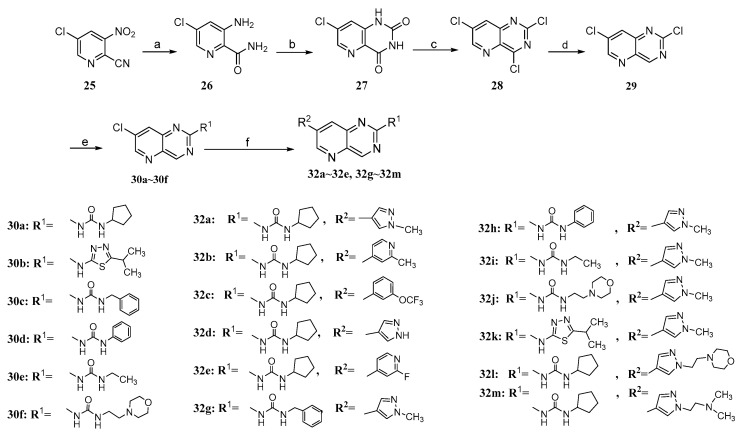
Preparation of target compounds **32a**~**32e** and **32g**~**32m**. Reagents and conditions: (**a**) SnCl_2_**·**2H_2_O, EtOH, reflux, 2 h; (**b**) Triphosgene, 1,4-dioxane, 110 °C, N_2_, 12 h; (**c**) POCl_3_, DIEA, 130 °C, N_2_; (**d**) Bu_3_SnH, Pd(PPh_3_)_4_, PhMe, N_2_, 100 °C, 1 h; (**e**) Cyclopentylurea (**14a**)/Benzylurea (**14b**)/Phenylurea (**14c**) Ethylurea (**14d**)/1-(2-morpholinoethyl)urea (**14e**)/5-methyl-1,3,4-thiadiazol-2-amine (**14f**), Pd(OAc)_2_, XantPhos, *t*-BuOK, 1,4-dioxane, 60 °C, N_2_, 2 h. (**f**) (2-methylpyridin-4-yl)boronic acid (**12a**)/1-methyl-4-(4,4,5,5-tetramethyl-1,3,2-dioxaborolan-2-yl)-1*H*-pyrazole (**12b**)/(3-(trifluoromethoxy) phenyl)boronic acid (**12c**)/(2-fluoropyridin-4-yl) boronic acid (**14g**)/4-(2-(4-(4,4,5,5-tetramethyl-1,3,2-dioxaborolan-2-yl)-1*H*-pyrazol-1-yl)ethyl) morpholine (**12d**)/*N*,*N*-dimethyl-2-(4-(4,4,5,5-tetramethyl-1,3,2-dioxaborolan-2-yl)-1*H*-pyrazol-1-yl)ethan-1-amine (**12e**), Pd(dppf)Cl_2_, K_2_CO_3_, V_(1,4-dioxane)_:V_(H2O)_ = 5:1, 60 °C, N_2_, 2 h.

**Scheme 4:**
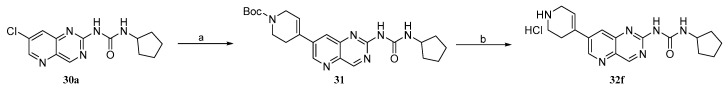
Preparation of target compounds **32f**. Reagents and conditions: (**a**) *tert*-butyl 4-(4,4,5,5-tetramethyl-1,3,2-dioxaborolan-2-yl)-3,6-dihydropyridine-1(2*H*)-carboxylate (**14h**), Pd(dppf)Cl_2_, K_2_CO_3_, V_(1,4-dioxane)_:V_(H2O)_ = 5:1, 60 °C, N_2_, 2 h; (**b**) EtOAc (HCl), EtOAC, rt, 2 h.

The synthetic routes of all target compounds are outlined in Scheme 1, Scheme 2, Scheme 3 and Scheme 4, respectively. Compounds **16a**~**16d** were synthesized using the synthetic route as shown in Scheme 1. Starting materials **7** was transformed into aldehyde **8** via Bouveault reaction. The resulting aldehyde **8** reacted with hydrazine hydrate to afford the cyclization intermediate **9** followed by reacting with NIS to gain the iodine intermediate **10**. TrtCl was added to protect the pyrazole N atom, and then **11** underwent the Suzuki C-C coupling reaction and Buchwald C-N coupling reaction to produce the intermediates **15a**~**15d**. After removing protect group Trt in the presence of F_3_CCO_2_H in DCM at room temperature (r.t) for 12 h, target compounds **16a**~**16d** were obtained.

The synthetic route of compound **24** was shown in Scheme 2. 2-Aminonicotinic acid **17** was transformed into amide **18** by condensation with ammonium chloride in the presence of DIEA and EDCI in DMF at r.t. for 12 h, followed by chlorination, cyclization with diphosgene and aromatization to afford the key intermediate **21**. C4-Cl of the key intermediate **21** was removed to gain **22** in the presence of Bu_3_SnH catalyzed by Pd(PPh_3_)_4_. Finally, **22** underwent the Suzuki C-C coupling reaction and Buchwald C-N coupling reaction to produce the target compound **24**.

As shown in Scheme 3, compounds **32a**~**32e** and **32g**~**32m** were synthesized from key intermediate **29**, undergoing Pd-catalyzed Suzuki C-C coupling reaction and Buchwald C-N coupling reaction. The intermediate **29** was synthesized from 3-amino-5-chloropicolinamide **26** through cyclization and aromatization.

The synthesis of target compound **32f** is described in Scheme 4. Substate 1-(7-chloropyrido[3,2-d]pyrimidin-2-yl)-3-cyclopentylurea **30a** was reacted with borate **14h** to afford the key intermediate **31** by catalysis of Pd(dppf)Cl_2_. Target compound **32f** was obtained after deprotected group Boc in the presence of HCl in EtOAc at r.t for 2 h. All compounds were characterized by ^1^H-NMR and ^13^C-NMR spectroscopy, HRMS and IR spectrometry for their structure and purity confirmation.

### 2.3. In Vitro ERK and PI3K Inhibition Assay

The compounds **16a**~**16d** were evaluated for ERK 2 and PI3Kα inhibitory activities in vitro. The results showed that compound **16b** had certain PI3Kα inhibitory activity (PI3Kα inhibition% (1000 nM) = 27.4%), while retaining ERK 2 inhibitory activity (ERK 2 inhibition% (1000 nM) = 84.8%) (Table 1).

In our efforts to improve the PI3K inhibitory activity of **16b**, the 1*H*-pyrazolo [3,4-*d*]pyrimidine scaffold was replaced by the pyrido[3,2-*d*]pyrimidine scaffold or pyrido[2,3-*d*]pyrimidine scaffold, leading to compound **24** and **32a**.

As shown in Table 2, **32a** with pyrido[3,2-*d*]pyrimidine scaffold exhibited good inhibitory activities on ERK2 and PI3Kα, while **24** with pyrido[2,3-*d*]pyrimidine scaffold had no inhibitory effect on ERK2 and PI3Kα. Consequently, we retained this pyrido[3,2-*d*]pyrimidine moiety for further modifications (Table 3). Firstly, we designed different R^1^ substituents to replace the 1-methyl-1*H*-pyrazol-4-yl. Five compounds (**32b**~**32f**) were synthesized and evaluated for ERK 2 and PI3Kα inhibitory activities. When R^1^ was pyrazolyl (**32d**), the inhibitory activity of ERK2 and PI3Kα kinases were retained to a certain extent. When R^1^ was 2-methylpyridin-4-yl (**32b**) or 2-fluoropyridin-4-yl (**32e**), the PI3Kα inhibitory activity was significantly reduced. Replacing R^1^ with 3-trifluoromethoxyphenyl (**32c**) or 1,2,3,6-tetrahydropyridin-4-yl (**32f**) will completely lose ERK2 and PI3Kα inhibitory activity. Second, we modified the structure of R^2^ and found that when R^2^ was benzylcarbamoyl (**32g**) or 2-isopropyl-1,3,4-thiazol-5-yl (**32k**), the inhibitory activity on PI3Kα was decreased and when R^2^ was phenylcarbamoyl (**32h**), ethylcarbamoyl (**32i**) or 2-morpholinoethylcarbamoyl (**32j**), the inhibitory activity on both kinases was subtracted.

Finally, compounds **32a**, **32d** and **32g** were obtained with dual inhibition of ERK2 and PI3Kα, and their IC_50_ values were evaluated, respectively (Table 4). Furthermore, to improve physicochemical properties, hydrophilic group ethylmorpholine or *N*,*N*-dimethylethyl was introduced to the NH of pyrazole, which closed to the solvent accessible area, so that compounds **32l** and **32m** were achieved. Their solubility was ameliorated while kinase inhibition experiments displayed that the introduction of larger hydrophilic groups to the NH of pyrazole was not conducive to the inhibitory activity of ERK2 (Table 3).

To investigate selectivity against ERK and PI3K isoforms, we also evaluated ERK 1, PI3Kβ, PI3Kγ and PI3Kδ inhibitory activities of compound **32d** (Table 5). The results showed that our compound also has good inhibitory effects on ERK1 and PI3Kγ.

### 2.4. In Vitro Anti-Proliferation Assay

Given sufficient potency in inhibitory activities against both ERK and PI3K kinases, **32a**, **32d**, **32g** and **32l** were chosen to evaluate for their anti-proliferative activities against HEC1B and HCT116 tumor cell lines. According to the results in Table 6, all the inhibitors showed moderate activities against HEC1B and HCT116. Among them, the proliferation inhibitory activity of all compounds on HEC1B was better than that of the positive compound BVD-523, and the inhibitory activity of compound **32d** on HCT116 was better than that of the positive compound GDC-0980.

### 2.5. In Vitro Pharmacokinetic (PK) Profile

To investigate in vitro pharmacokinetic characteristics, **32d** was selected to be incubated at 90 ng/mL in human liver microsomes (Table 7). The results showed that **32d** possessed a relatively long half-life of 173.25 min and a moderate clearance rate (0.016 mL/min/mg) indicating compound **32d** had good metabolic stability in vitro.

### 2.6. In Vivo Pharmacokinetic (PK) Profile

Compound **32d** was selected to further investigate in vivo pharmacokinetic characteristics. The compound was administrated intravenously (i.v.) at 1 mg/kg or orally (p.o.) at 10 mg/kg in Sprague Dawley (SD) rats. The pharmacokinetic parameters of **32d** were shown in Table 8, which showed that **32d** possessed the unsatisfactory oral bioavailability (F) of 9.37% and the moderate half-life of intravenous administration (t_1/2_ = 2.32 h). Therefore, it indicated that the intravenous administration was superior to oral gavage, insuring further evaluation of its intravenous antitumor activity once a day.

### 2.7. In Vivo Antitumor Activity Evaluation

**Figure 6:**
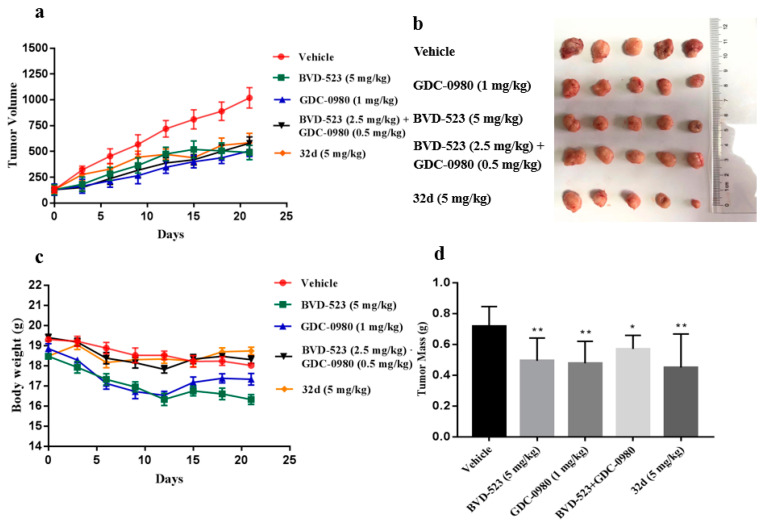
Antitumor efficacy of **32d** in the HCT-116 mouse xenograft model. (**a**) HCT-116 mousexenograft data with compound **32d**. (**b**) The resulting tumors were excised from the indicated groups. (**c**) The nude mouse body weight was measured every 3 days. (**d**) The tumor masses for five groups of animals were compared, and each histogram represents the Mean ± SD of 6 mice. * *p* < 0.1, ** *p* < 0.05 vs. control.

Based on the good enzymatic and antiproliferative activities of compound **32d** in vitro, we then evaluated its antitumor activities in vivo in nude mice with HCT-116 xenograft. Compounds **32d** (5 mg/kg), BVD-523 (5 mg/kg), GDC-0980 (1 mg/kg), and the combination of BVD-523 and GDC-0980 (2.5 mg/kg + 0.5 mg/kg) were administered by intraperitoneal injection once daily (BID) for 21 consecutive days. The body weight and tumor size of mice were measured every 3 days. As shown in Figure 6, although the tumor suppression effects of **32d** (TGI: 51%) was equal to positive compound BVD-523 (TGI: 50%) and more effective than GDC-0980 (TGI: 46%) and the combination of BVD-523 and GDC-0980 (TGI: 45%), there was no observed body weight loss with treatment by 21 days in this study (Figure S2), which proved good safety of **32d**. In comparison, due to the serious toxicity of positive compounds BVD-523 and GDC-0980, the body weight of nude mice with the treatment of them was losing.

## 3. Materials and Methods

### 3.1. Synthesis

Unless otherwise stated, all reagents were purchased from commercial suppliers and used without purifications. ^1^H-NMR and ^13^C-NMR spectra were obtained on a BRUKER AVANCE AV-300 nuclear instrument (Beijing, China) in CDCl_3_ or DMSO-*d*_6_ using TMS as internal standard, operating at 300 MHz and 75 MHz, respectively. Chemical shifts (*δ*) are expressed in ppm and coupling constants J are given in Hz. Analytical thin layer chromatography (TLC) was performed on pre-coated, glass-backed silica gel plates. Visualization of the developed chromatogram was performed by UV absorbance (254 nm). High resolution mass spectra (HRMS) were obtained on a Waters Q-TOF micro TM apparatus (Milford, MA, USA). Melting points were measured with an RY-I melting point apparatus.

#### 3.1.1. 2,4-Dichloropyrimidine-5-carbaldehyde (**8**)

To a solution of 5-bromo-2,4-dichloropyrimidine **7** (27.95 mmol) in dry THF (30 mL) was charged with N_2_ and added isopropylmagnesium chloride lithium chloride complex (1.3 M in THF, 21.5 mL, 27.95 mmol) at −78 °C and stirred for 2 h. Then dry DMF (4.4 mL, 55.90 mmol) was dropped into the solution slowly and allowed to −42 °C for 12h. After completion (monitored by TLC), 1 N HCl (44 mL) was added and extracted three times with diethyl ether. The combined organic extracts was dried (Na_2_SO_4_), concentrated under reduced pressure and the residue was purification by column chromatography on silica gel to give a white solid. Yield 38%, m.p. 74–76 °C. ^1^H-NMR (300 MHz, CDCl_3_) *δ* (ppm): 10.39 (s, 1H, CHO), 9.01 (s, 1H, ArH).

#### 3.1.2. 6-Chloro-1*H*-pyrazolo[3,4-*d*]pyrimidine (**9**)

To a solution of 2,4-dichloropyrimidine-5-carbaldehyde **8** (10.31 mmol) in THF (5 mL), 80% hydrazine hydrate (20.62 mmol) at 0 °C was added and stirred for 1 h. After completion (monitored by TLC), suction and the filtrate was extracted three times with DCM. The combined organic extracts was dried (Na_2_SO_4_), concentrated under reduced pressure and the residue was purification by column chromatography on silica gel to give a yellow solid. Yield 44%, m.p. 188–189 °C. ^1^H-NMR (300 MHz, DMSO-*d*_6_) *δ* (ppm): 14.31 (s, 1H, NH), 9.31 (s, 1H, ArH), 8.45 (s, 1H, ArH).

#### 3.1.3. 6-Chloro-3-iodo-1*H*-pyrazolo[3,4-*d*]pyrimidine (**10**)

A solution of 6-chloro-1*H*-pyrazolo[3,4-*d*]pyrimidine **9** (1.29 mmol) and NIS (1.55 mmol) in DMF (3 mL) was stirred at r.t for 12 h. After completion (monitored by TLC), water (10 mL) was added and the mixture was extracted three times with EA. The combined organic extracts was dried (Na_2_SO_4_), concentrated under reduced pressure and the residue was purification by column chromatography on silica gel to give a white solid. Yield 56%, m.p. 237–238 °C. ^1^H-NMR (300 MHz, DMSO-*d*_6_) *δ* (ppm): 11.00 (s, 1H, NH), 6.41 (s, 1H, ArH).

#### 3.1.4. 6-Chloro-3-iodo-1-trityl-1*H*-pyrazolo[3,4-*d*]pyrimidine (**11**)

A solution of 6-chloro-3-iodo-1*H*-pyrazolo[3,4-*d*] pyrimidine **10** (3.98 mmol) and K_2_CO_3_ (7.96 mmol) in MeCN (10 mL) was added TrtCl (3.98 mmol) at 0 °C and stirred at r.t for 16 h. After completion (monitored by TLC), water (15 mL) was added and the mixture was extracted three times with EA. The combined organic extracts were dried (Na_2_SO_4_), concentrated under reduced pressure and the residue was purification by column chromatography on silica gel to give a white solid. Yield 67%, m.p. 127–129 °C. ^1^H-NMR (300 MHz, CDCl_3_) *δ* (ppm): 8.72 (s, 1H, ArH), 7.37–7.32 (m, 9H, ArH), 7.29–7.23 (m, 6H, ArH).

#### 3.1.5. General Procedure for the Synthesis of **13a**, **13b**

A solution of 6-chloro-3-iodo-1-trityl-1*H*-pyrazolo[3,4-*d*]pyrimidine **11** (0.77 mmol), appropriate borate or boric acid (0.77 mmol), Pd(dppf)Cl_2_ (0.077 mmol) and K_2_CO_3_ (1.53 mmol) in mixed-solvent (5 mL, *V*_dioxane_:*V*_H2O_ = 5:1) was charged with N_2_ and stirred at 60 °C for 6 h. After completion (monitored by TLC), cooled to r.t, concentrated under reduced pressure and the residue was purification by column chromatography on silica gel to give product **13a**, **13b**.

##### 6-Chloro-3-(2-methylpyridin-4-yl)-1-trityl-1*H*-pyrazolo[3,4-*d*]pyrimidine (**13a**)

Yellow solid. Yield 53%, m.p. 220–222 °C. ^1^H-NMR (300 MHz, DMSO-*d*_6_) *δ* (ppm): 9.77 (s, 1H, ArH), 8.60 (d, *J* = 5.2 Hz, 1H, ArH), 7.85 (s, 1H, ArH), 7.72 (dd, *J* = 5.3, 1.7 Hz, 1H, ArH), 7.43–7.24 (m, 15H, TrtCl-H), 2.59 (s, 3H, CH_3_).

##### 6-Chloro-3-(1-methyl-1*H*-pyrazol-4-yl)-1-trityl-1*H*-pyrazolo[3,4-*d*]pyrimidine (**13b**)

Light-yellow solid. Yield 73%, m.p. 228–230 °C. ^1^H-NMR (300 MHz, DMSO-*d*_6_) *δ* (ppm): 9.50 (s, 1H, ArH), 8.39 (s, 1H, ArH), 8.01 (d, *J* = 0.8 Hz, 1H, ArH), 7.35–7.28 (m, 9H, TrtCl-H), 7.25–7.22 (m, 6H, TrtCl-H), 3.91 (s, 3H, NCH_3_).

#### 3.1.6. General Procedure for the Synthesis of **15a**~**15d**

A solution of aryl chloride (0.21 mmol), amino compound (0.23 mmol), Pd_2_(dba)_3_ (0.02 mmol), XantPhos (0.02 mmol) and Cs_2_CO_3_ (0.42 mmol) in toluene (3 mL) was charged with N_2_ and stirred at 110 °C for 6 h. After completion (monitored by TLC), cooled to r.t, concentrated under reduced pressure and the residue was purification by column chromatography on silica gel to give product **15a**~**15d**.

##### 1-Benzyl-3-(3-(2-methylpyridin-4-yl)-1-trityl-1*H*-pyrazolo[3,4-*d*]pyrimidin-6-yl)urea (**15a**)

Yellow solid. Yield 97%, m.p. >250 °C. ^1^H-NMR (300 MHz, DMSO-*d*_6_) *δ* (ppm): 10.19 (s, 1H, NH), 9.59 (s, 1H, ArH), 8.59 (d, *J* = 5.4 Hz, 1H, ArH), 8.25 (t, *J* = 6.3 Hz, 1H, NH), 7.81 (s, 1H, ArH), 7.69 (d, *J* = 5.6 Hz, 1H, ArH), 7.34–7.32 (m, 3H, ArH), 7.29–7.27 (m, 9H, TrtCl-H), 7.23–7.20 (m, 6H, TrtCl-H), 7.03 (d, *J* = 6.8 Hz, 2H, ArH), 4.20 (d, *J* = 6.0 Hz, 2H, CH_2_Ph), 2.59 (s, 3H, CH_3_).

##### 1-Cyclopentyl-3-(3-(2-methylpyridin-4-yl)-1-trityl-1*H*-pyrazolo[3,4-*d*]pyrimidin-6-yl)urea (**15b**)

Yellow solid. Yield 97%, m.p. 228–230 °C. ^1^H-NMR (300 MHz, CDCl_3_) *δ* (ppm): 9.29 (s, 1H, ArH), 8.63 (d, *J* = 5.4 Hz, 1H, ArH), 7.73–7.69 (m, 2H, ArH), 7.37–7.32 (m, 9H, TrtCl-H), 7.24–7.21 (m, 6H, TrtCl-H), 4.00–3.92 (m, 1H, NHCH), 2.78 (s, 3H, CH_3_), 1.89–1.83 (m, 2H, cyclopentane-H), 1.62–1.56 (m, 6H, cyclopentane-H).

##### 1-Benzyl-3-(3-(1-methyl-1*H*-pyrazol-4-yl)-1-trityl-1*H*-pyrazolo[3,4-*d*]pyrimidin-6-yl)urea (**15c**)

Brown solid. Yield 94%, m.p. >250 °C. ^1^H-NMR (400 MHz, DMSO-*d*_6_) *δ* (ppm): 10.04 (s, 1H, NH), 9.32 (s, 1H, ArH), 8.30–8.25 (m, 2H, ArH, NH), 7.96 (s, 1H, ArH), 7.36–7.28 (m, 3H, ArH), 7.34–7.19 (m, 15H, TrtCl-H), 7.02 (d, *J* = 7.1 Hz, 2H, ArH), 3.92–3.90 (m, 5H, CH_2_Ph, CH_3_).

##### 1-Cyclopentyl-3-(3-(1-methyl-1*H*-pyrazol-4-yl)-1-trityl-1*H*-pyrazolo[3,4-*d*]pyrimidin-6-yl)urea (**15d**)

Light-yellow solid. Yield 89%, m.p. >250 °C. ^1^H-NMR (400 MHz, CDCl_3_) *δ* (ppm): 9.03 (s, 1H, ArH), 7.90 (s, 1H, ArH), 7.78 (s, 1H, ArH), 7.30–7.28 (m, 9H, TrtCl-H), 7.23–7.21 (m, 6H, TrtCl-H), 3.98–3.91 (m, 4H, CH3, NHCH), 1.88–1.79 (m, 2H, cyclopentane-H), 1.59–1.49 (m, 6H, cyclopentane-H).

#### 3.1.7. 2-Aminonicotinamide (**18**)

To a solution of 2-aminonicotinic acid **17** (21.72 mmol), EDCI (32.58 mmol), HOBt (32.58 mmol) and DIEA (65.16 mmol) in DMF (10 mL) was added ammonium chloride (43.44 mmol); the mixture was then stirred at r.t for 12 h. After completion (monitored by TLC), water (10 mL) was added and extracted three times with ethyl acetate. The combined organic extracts were dried (Na_2_SO_4_) and concentrated under reduced pressure to give a white solid. Yield 95%, m.p. 183–184 °C. ^1^H-NMR (300 MHz, DMSO-*d*_6_) δ (ppm): 8.06 (dd, *J* = 4.8, 1.8 Hz, 1H, ArH), 7.94 (dd, *J* = 7.7, 1.9 Hz, 2H, ArH, CONH_2_), 7.35–7.28 (m, 1H, CONH_2_), 7.19 (s, 2H, NH_2_), 6.55 (dd, *J* = 7.7, 4.8 Hz, 1H).

#### 3.1.8. 2-Amino-5-chloronicotinamide (**19**)

To a solution of 2-aminonicotinamide **18** (160.42 mmol) in conc. HCl (150 mL) was added 30% H_2_O_2_ (320.84 mmol), then the mixture was allowed to 60 °C and stirred for 6 h. After completion (monitored by TLC), it was cooled to r.t and concentrated under reduced pressure to remove the solvent. Then pH was adjusted to 9~10 at 0 °C and the precipitation was separated out. Suction and drying to give a gray solid. Yield 36%, m.p. 240–242 °C. ^1^H-NMR (300 MHz, DMSO-*d*_6_) *δ* (ppm): 8.10–8.06 (m, 3H, ArH, CONH_2_), 7.47 (s, 1H, CONH_2_), 7.36 (s, 2H, NH_2_). 1H NMR (300 MHz, DMSO-*d*_6_-D_2_O) *δ* (ppm): 8.09 (d, *J* = 2.6 Hz, 1H, ArH), 8.03 (d, *J* = 2.5 Hz, 1H, ArH).

#### 3.1.9. 6-Chloropyrido[2,3-*d*]pyrimidine-2,4(1*H*,3*H*)-dione (**20**)

To a solution of 2-amino-5-chloronicotinamide **19** (34.97 mmol) in 1,4-dioxane (30 mL) was added diphosgene (111.90 mmol), then the mixture was charged with N_2_ and stirred at 120 °C for 12 h. After completion (monitored by TLC), cooled to r.t. Dry diethyl ether (100 mL) was added and stirred at r.t for 1 h, the precipitation separated out. Suction and drying to give a brown solid. Yield 71%, m.p. >250 °C. ^1^H-NMR (300 MHz, DMSO-*d*_6_) *δ* (ppm): 11.88 (s, 1H, NH), 11.62 (s, 1H, NH), 8.66 (d, *J* = 2.6 Hz, 1H, ArH), 8.26 (d, *J* = 2.5 Hz, 1H, ArH).

#### 3.1.10. 2,4,6-Trichloropyrido[2,3-*d*]pyrimidine (**21**)

To a solution of 6-chloropyrido[2,3-*d*] pyrimidine-2,4(1*H*,3*H*)-dione **20** (0.51 mmol) and DIEA (1.02 mmol) in phosphorus oxychloride (30 mL), then the mixture was charged with N_2_ and stirred at 130 °C for 12 h. After completion (monitored by TLC), cooled to r.t and concentrated under reduced pressure to remove the solvent. Water (3 mL) was added to quench the reaction at 0 °C. Then, it was extracted three times with DCM and the combined organic extracts were dried (Na_2_SO_4_) and concentrated under reduced pressure; the residue was purified by column chromatography on silica gel to give a white solid. Yield 36%, m.p. 138–140 °C. ^1^H-NMR (300 MHz, DMSO-*d*_6_) *δ* (ppm): 8.63 (d, *J* = 2.5 Hz, 1H, ArH), 8.26 (d, *J* = 2.5 Hz, 1H, ArH).

#### 3.1.11. 2,6-Dichloropyrido[2,3-*d*]pyrimidine (**22**)

To a solution of 2,4,6-trichloropyrido[2,3-*d*]pyrimidine **21** (0.30 mmol) and Bu_3_SnH (0.30 mmol) and Pd(PPh_3_)_4_ (0.02 mmol) in toluene (2 mL), then the mixture was charged with N_2_ and stirred at 100 °C for 1 h. After completion (monitored by TLC), cooled to r.t and concentrated under reduced pressure, the residue was purified by column chromatography on silica gel to give a white solid. Yield 53%, m.p. 224–226 °C. ^1^H-NMR (300 MHz, DMSO-*d*_6_) *δ* (ppm): 9.71 (s, 1H, ArH), 9.37 (d, *J* = 2.8 Hz, 1H, ArH), 8.94 (d, *J* = 2.7 Hz, 1H, ArH).

#### 3.1.12. 1-(6-Chloropyrido[2,3-*d*]pyrimidin-2-yl)-3-cyclopentylurea (**23**)

To a solution of Pd(OAc)_2_ (0.19 mmol) and XantPhos (0.28 mmol) in 1,4-dioxane (2 mL) was charged with N_2_ and stirred at r.t for 1 h. After that 2,6-dichloropyrido[2,3-*d*]pyrimidine **22** (0.94 mmol) and 1-cyclopentylurea (0.94 mmol) and potassium tert-butoxide (1.40 mmol) was added to the solution, then the mixture was charged with N_2_ and stirred at 60 °C for 2 h. After completion (monitored by TLC), cooled to r.t and concentrated under reduced pressure, the residue was purified by column chromatography on silica gel to give a yellow solid. Yield 31%, m.p. 175–177 °C. ^1^H-NMR (300 MHz, DMSO-*d*_6_) *δ* (ppm): 10.36 (s, 1H, NH), 9.63 (d, *J* = 7.6 Hz, 1H, NH), 9.50 (s, 1H, ArH), 9.14 (d, *J* = 2.8 Hz, 1H, ArH), 8.69 (d, *J* = 2.8 Hz, 1H, ArH), 4.18–4.12 (m, 1H, NHCH), 2.02–1.94 (m, 2H, cyclopentyl-H), 1.80–1.73 (m, 2H, cyclopentyl), 1.68–1.63 (m, 2H, cyclopentyl-H), 1.58–1.54 (m, 2H, cyclopentyl-H).

#### 3.1.13. 3-Amino-5-chloropicolinamide (**26**)

To a solution of 5-chloro-3-nitropicolinonitrile **25** (108.96 mmol) in EtOH (100 mL) was added SnCl_2_**·**2H_2_O (435.85 mmol) in portions at 0 °C. The mixture was allowed to 78 °C and stirred for 2 h. After completion (monitored by TLC), cooled to r.t and concentrated under reduced pressure, the residue was dissolved in water (100 mL): ultrasonic concussion. The pH was adjusted to 9~10 with 6 M NaOH aq. and the mixture was extracted three times with ethyl acetate. The combined organic extracts were dried (Na_2_SO_4_) and concentrated under reduced pressure to give a white solid. Yield 61%, m.p. 156–158 °C. ^1^H-NMR (300 MHz, DMSO-*d*_6_) *δ* (ppm): 7.93 (s, 1H, CONH_2_), 7.76 (d, *J* = 2.1 Hz, 1H, ArH), 7.45 (s, 1H, CONH_2_), 7.27 (d, *J* = 2.1 Hz, 1H, ArH), 7.11 (s, 2H, NH_2_).

#### 3.1.14. 7-Chloropyrido[3,2-*d*]pyrimidine-2,4(1*H*,3*H*)-dione (**27**)

To a solution of 3-amino-5-chloropicolinamide **26** (66.70 mmol) and triphosgene (66.70 mmol) in 1,4-dioxane (100 mL) was charged with N_2_ and stirred at 110 °C for 12 h. After completion (monitored by TLC) and being cooled to at 0 °C, water (100 mL) was added and stirred for 30 min to quench the reaction. Suction and filter cake were washed with water. It was then dried to give the light green solid. Yield 95%, m.p. >250 °C. ^1^H-NMR (300 MHz, DMSO-*d*_6_) *δ* (ppm): 11.62 (s, 1H, NH), 11.35 (s, 1H, NH), 8.49 (d, *J* = 2.1 Hz, 1H, ArH), 7.62 (d, *J* = 2.2 Hz, 1H, ArH).

#### 3.1.15. 2,4,7-Trichloropyrido[3,2-d]pyrimidine (**28**)

In a solution of 7-chloropyrido[3,2-*d*]pyrimidine-2,4(1*H*,3*H*)-dione **27** (63.42 mmol) and DIEA (126.84 mmol) in phosphorus oxychloride (30 mL), the mixture was charged with N_2_ and stirred at 130 °C for 12 h. After completion (monitored by TLC), it was cooled to r.t and concentrated under reduced pressure to remove the solvent. Water (3 mL) was added to quench the reaction at 0 °C. Then, it was extracted three times with EA and the combined organic extracts were dried (Na_2_SO_4_), concentrated under reduced pressure and the residue was purified by column chromatography on silica gel to give a white solid. Yield 66%, m.p. >250 °C. ^1^H-NMR (300 MHz, DMSO-*d*_6_) *δ* (ppm): 8.84 (d, *J* = 2.2 Hz, 1H, ArH), 8.29 (d, *J* = 2.2 Hz, 1H, ArH).

#### 3.1.16. 2,7-Dichloropyrido[3,2-*d*]pyrimidine (**29**)

To a solution of 2,4,7-trichloropyrido[3,2-*d*]pyrimidine **27** (2.13 mmol) and Bu_3_SnH (2.13 mmol) and Pd(PPh_3_)_4_ (0.11 mmol) in toluene (10 mL), then the mixture was charged with N_2_ and stirred at 100 °C for 1 h. After completion (monitored by TLC), cooled to r.t, concentrated under reduced pressure and the residue was purification by column chromatography on silica gel to give a pink solid. Yield 74%, m.p. 180–181 °C. ^1^H-NMR (300 MHz, CDCl_3_) *δ* (ppm): 9.56 (s, 1H, ArH), 9.03 (d, *J* = 2.3 Hz, 1H, ArH), 8.32 (d, *J* = 2.3 Hz, 1H, ArH).

#### 3.1.17. General Procedure for the Synthesis of **30a**~**30f**

A solution of Pd(OAc)_2_ (0.19 mmol) and XantPhos (0.28 mmol) in 1,4-dioxane (2 mL) was charged with N_2_ and stirred at r.t for 1 h. After that 2,7-dichloropyrido[3,2-d]pyrimidine **29** (0.94 mmol) and appropriate amino compound (0.94 mmol) and potassium tert-butoxide (1.40 mmol) was added to the solution; then, the mixture was charged with N_2_ and stirred at 60 °C for 2 h. After completion (monitored by TLC), it was cooled to r.t and concentrated under reduced pressure and the residue was purified by column chromatography on silica gel to give the product **30a**~**30f**.

##### 1-(7-Chloropyrido[3,2-*d*]pyrimidin-2-yl)-3-cyclopentylurea (**30a**)

Pink solid. Yield 54%, m.p. 212–214 °C. ^1^H-NMR (300 MHz, DMSO-*d*_6_) *δ* (ppm): 10.33 (s, 1H, NH), 9.50 (s, 1H, ArH), 9.29 (d, *J* = 7.1 Hz, 1H, NH), 8.91 (d, *J* = 2.2 Hz, 1H, ArH), 8.51 (d, *J* = 2.3 Hz, 1H, ArH), 4.13 (q, *J* = 6.5 Hz, 1H, NHCH), 2.01–1.96 (m, 2H, cyclopentyl-H), 1.81–1.73 (m, 3H, cyclopentyl-H), 1.67–1.63 (m, 3H, cyclopentyl-H).

##### *N*-(7-chloropyrido[3,2-*d*]pyrimidin-2-yl)-5-methyl-1,3,4-thiadiazol-2-amine (**30b**)

Yellow solid. Yield 70%, m.p. >250 °C. ^1^H-NMR (300 MHz, DMSO-*d*_6_) *δ* (ppm): 12.56 (s, 1H, NH), 9.55 (s, 1H, ArH), 8.90 (d, *J* = 2.3 Hz, 1H, ArH), 8.41 (d, *J* = 2.2 Hz, 1H, ArH), 2.68 (s, 3H, CH_3_).

##### 1-Benzyl-3-(7-chloropyrido[3,2-*d*]pyrimidin-2-yl)urea (**30c**)

Light-yellow solid. Yield 27%, m.p. 191–192 °C. ^1^H-NMR (300 MHz, DMSO-*d*_6_) *δ* (ppm): 10.44 (s, 1H, NH), 9.82 (t, *J* = 6.1 Hz, 1H, NH), 9.49 (d, *J* = 0.8 Hz, 1H, ArH), 8.89 (d, *J* = 2.2 Hz, 1H, ArH), 8.56–8.55 (m, 1H, ArH), 7.39–7.32 (m, 5H, Ph-H), 4.53 (d, *J* = 6.1 Hz, 2H, CH_2_Ph).

##### 1-(7-Chloropyrido[3,2-*d*]pyrimidin-2-yl)-3-phenylurea (**30d**)

Light-yellow solid. Yield 35%, m.p. >238–240 °C. ^1^H-NMR (300 MHz, DMSO-*d*_6_) *δ* (ppm): 11.64 (s, 1H, NH), 10.76 (s, 1H, NH), 9.57 (d, *J* = 0.8 Hz, 1H, ArH), 8.95 (d, *J* = 2.2 Hz, 1H, ArH), 8.82 (dd, *J* = 2.2, 0.8 Hz, 1H, ArH), 7.82–7.79 (m, 2H, Ph-H), 7.38 (t, *J* = 7.9 Hz, 2H, Ph-H), 7.11 (t, *J* = 7.4 Hz, 1H, Ph-H).

##### 1-(7-Chloropyrido[3,2-*d*]pyrimidin-2-yl)-3-ethylurea (**30e**)

Light-yellow solid. Yield 20%, m.p. 208–210 °C. ^1^H-NMR (300 MHz, DMSO-*d*_6_) *δ* (ppm): 10.29 (s, 1H, NH), 9.47 (d, *J* = 0.8 Hz, 1H, ArH), 9.30 (t, *J* = 5.6 Hz, 1H, NH), 8.88 (d, *J* = 2.3 Hz, 1H, ArH), 8.59 (dd, *J* = 2.3, 0.8 Hz, 1H, ArH), 3.32–3.27 (m, 2H, CH_2_CH_3_), 1.19 (t, *J* = 7.2 Hz, 3H, CH_2_CH_3_).

##### 1-(7-Chloropyrido[3,2-*d*]pyrimidin-2-yl)-3-(2-morpholinoethyl)urea (**30f**)

Light-brown solid. Yield 48%, m.p. 188–189 °C. ^1^H-NMR (300 MHz, DMSO-*d*_6_) *δ* (ppm): 9.47–9.46 (m,1H, CONH), 9.44 (s, 1H, ArH), 8.81 (d, *J* = 2.2 Hz, 1H, ArH), 8.20–8.17 (m, 1H, CONH), 8.15 (d, *J* = 2.3 Hz, 1H, ArH), 3.85 (d, *J* = 4.5 Hz, 4H, O(CH_2_)_2_), 3.64 (q, *J* = 5.8 Hz, 2H, NHCH_2_), 2.72 (t, *J* = 6.1 Hz, 2H, NCH_2_), 2.65 (t, *J* = 4.8 Hz, 4H, N(CH_2_)_2_).

#### 3.1.18. General Procedure for the Synthesis of **16a**~**16d**

To a solution of Trt-protected compound (0.20 mmol) in DCM (1.5 mL), trifluoroacetic acid was added and stirred at r.t for 12 h. After completion (monitored by TLC), the mixture was concentrated under reduced pressure and the residue was purified by refining (*V*_DCM_:*V*_MeOH_ = 20:1) to give the product **16a**~**16d**.

##### 1-Benzyl-3-(3-(2-methylpyridin-4-yl)-1*H*-pyrazolo[3,4-*d*]pyrimidin-6-yl)urea (**16a**)

White solid. Yield 93%, m.p. 228–230 °C. ^1^H-NMR (300 MHz, DMSO-*d*_6_) *δ* (ppm): 14.54 (s, 1H, NH), 10.26 (s, 1H, NH), 9.72 (s, 1H, ArH), 9.44 (t, *J* = 6.0 Hz, 1H, NH), 8.80 (d, *J* = 6.1 Hz, 1H, ArH), 8.43 (d, *J* = 1.7 Hz, 1H, ArH), 8.34 (dd, *J* = 6.0, 1.8 Hz, 1H, ArH), 7.37 (d, *J* = 4.4 Hz, 4H, Ph-H), 7.32–7.24 (m, 1H, Ph-H), 4.53 (d, *J* = 5.9 Hz, 2H, NHCH_2_), 2.79 (s, 3H, CH_3_). ^13^C-NMR (75 MHz, Acetic-*d*_4_) *δ* (ppm): 156.07, 155.81, 147.82, 145.25, 142.02, 140.25, 140.24, 138.35, 128.59, 127.27, 127.22, 121.16, 119.13, 113.09, 108.16, 43.62, 12.76. HRMS (ESI): *m*/*z* [M + H]^+^. Calcd for C_19_H_18_N_7_O: 360.1567; Found: 360.1571.

##### 1-Cyclopentyl-3-(3-(2-methylpyridin-4-yl)-1*H*-pyrazolo[3,4-*d*]pyrimidin-6-yl)urea (**16b**)

White solid. Yield 90%, m.p. 210–212 °C. ^1^H-NMR (300 MHz, DMSO-*d*_6_) *δ* (ppm): 14.37 (s, 1H, NH), 10.03 (s, 1H, NH), 9.68 (s, 1H, ArH), 9.07 (d, *J* = 7.0 Hz, 1H, NH), 8.76 (d, *J* = 5.8 Hz, 1H, ArH), 8.29 (s, 1H, ArH), 8.21 (d, *J* = 5.9 Hz, 1H, ArH)., 4.14–4.08 (m, 1H, NHCH), 2.73 (s, 3H, CH_3_), 1.99–1.90 (m, 2H, cyclopentyl-H), 1.74–1.49 (m, 6H, cyclopentyl-H). ^13^C-NMR (75 MHz, Acetic-*d*_4_) *δ* (ppm): 156.84, 156.25, 156.02, 155.06, 153.67, 145.58, 143.93, 140.38, 123.29, 120.36, 107.62, 51.60, 33.37, 23.73, 20.88. HRMS (ESI): *m*/*z* [M + H]^+^. Calcd for C_17_H_20_N_7_O: 338.1724; Found: 338.1729.

##### 1-Benzyl-3-(3-(1-methyl-1*H*-pyrazol-4-yl)-1*H*-pyrazolo[3,4-*d*]pyrimidin-6-yl)urea (**16c**)

White solid. Yield 65%, m.p. >250 °C. ^1^H-NMR (400 MHz, DMSO-*d*_6_) *δ* (ppm): 13.48 (s, 1H,NH), 10.03 (s, 1H, NH), 9.56 (t, *J* = 6.0 Hz, 1H, NH), 9.36 (s, 1H, ArH), 8.45 (s, 1H, ArH), 8.06 (s, 1H, ArH), 7.37–7.33 (m, 3H, ArH), 7.29–7.20 (m, 2H, ArH), 4.52 (d, *J* = 5.9 Hz, 2H, CH_2_Ph), 3.92 (s, 3H, CH_3_). ^13^C-NMR (75 MHz, DMSO-*d*_6_) *δ* (ppm): 156.63, 155.34, 154.72, 154.06, 140.16, 138.96, 137.22, 129.72, 128.95, 127.44, 127.35, 114.07, 107.58, 43.26, 39.20. HRMS (ESI): *m*/*z* [M + Na]^+^ Calcd for C_17_H_16_N_8_ONa: 371.1339; Found: 371.1346.

##### 1-Cyclopentyl-3-(3-(1-methyl-1*H*-pyrazol-4-yl)-1*H*-pyrazolo[3,4-*d*]pyrimidin-6-yl)urea (**16d**)

White solid. Yield 71%, m.p. >250 °C. ^1^H-NMR (300 MHz, DMSO-*d*_6_) *δ* (ppm): 13.48 (s, 1H, NH), 9.81 (s, 1H, NH), 9.36 (s, 1H, ArH), 9.18 (d, *J* = 7.0 Hz, 1H, NH), 8.47 (s, 1H, ArH), 8.07 (d, *J* = 0.8 Hz, 1H, ArH), 4.10 (p, *J* = 6.4 Hz, 1H, NHCH), 3.93 (s, 3H, NCH_3_), 1.97–1.89 (m, 2H, cyclopentyl-H), 1.82–1.40 (m, 6H, cyclopentyl-H). ^13^C-NMR (75 MHz, DMSO-*d*_6_) *δ* (ppm): 156.62, 155.17, 154.05, 153.91, 138.98, 137.20, 129.75, 114.07, 107.40, 51.54, 39.19, 33.39, 23.70. HRMS (ESI): *m*/*z* [M + H]^+^ Calcd for C_15_H_18_N_8_O: 327.1676; Found: 327.1681.

#### 3.1.19. General Procedure for the Synthesis of **24**, **32a**~**32e** and **32g**~**32m**

This general procedure is the same as the synthesis of **13a** and **13b**.

##### 1-Cyclopentyl-3-(6-(1-methyl-1*H*-pyrazol-4-yl)pyrido[2,3-*d*]pyrimidin-2-yl)urea (**24**)

White solid. Yield 81%, m.p. >250 °C. ^1^H-NMR (300 MHz, DMSO-*d*_6_) *δ* (ppm): 10.19 (s, 1H, NH), 9.73 (d, *J* = 7.1 Hz, 1H, NH), 9.46 (s, 1H, ArH), 9.42 (d, *J* = 2.6 Hz, 1H, ArH), 8.60 (d, *J* = 2.6 Hz, 1H, ArH), 8.40 (s, 1H, ArH), 8.10 (s, 1H, ArH), 4.19–4.13 (m, 1H, NHCH), 3.95 (s, 3H, NCH_3_), 2.00–1.94 (m, 2H, cyclopentyl-H), 1.81–1.77 (m, 2H, cyclopentyl-H), 1.69–1.54 (m, 4H, cyclopentyl-H). ^13^C-NMR (75 MHz, Aectic-*d*_4_) *δ* (ppm): 173.41, 168.76, 158.50, 154.79, 149.61, 128.97, 126.03, 123.00, 120.05, 112.91, 111.50, 64.04, 52.24, 32.57, 23.28. HRMS (ESI): *m*/*z* [M + H]^+^ Calcd for C_17_H_20_N_7_O: 338.1724; Found: 338.1727.

##### 1-Cyclopentyl-3-(6-(1-methyl-1*H*-pyrazol-4-yl)pyrido[2,3-*d*]pyrimidin-2-yl)urea (**32a**)

White solid. Yield 74%, m.p. >250 °C. ^1^H-NMR (300 MHz, DMSO-*d*_6_) *δ* (ppm): 10.14 (s, 1H, NH), 9.43 (d, *J* = 7.1 Hz, 1H, NH), 9.38 (s, 1H, ArH), 9.22 (s, 1H, ArH), 8.64 (s, 1H, ArH), 8.33 (s, 1H, ArH), 8.23 (s, 1H, ArH), 4.14–4.12 (m, 1H, NHCH), 3.97 (s, 3H, NCH_3_), 2.05–1.97 (m, 2H, cyclopentyl-H), 1.82–1.76 (m, 2H, cyclopentyl-H), 1.68–1.54 (m, 4H, cyclopentyl-H). ^13^C-NMR (75 MHz, Aectic-*d*_4_) *δ* (ppm): 161.86, 155.45, 155.42, 147.86, 146.61, 137.14, 134.64, 134.30, 130.19, 127.84, 118.02, 52.04, 38.28, 32.76, 23.30. HRMS (ESI): *m*/*z* [M + H]^+^ Calcd for C_17_H_20_N_7_O: 338.1724; Found: 338.1728.

##### 1-Cyclopentyl-3-(7-(2-methylpyridin-4-yl)pyrido[3,2-*d*]pyrimidin-2-yl)urea (**32b**)

White solid. Yield 66%, m.p. 240–242 °C. ^1^H-NMR (300 MHz, DMSO-d_6_) δ (ppm): 10.31 (s, 1H, NH), 9.56 (s, 1H, ArH), 9.39 (d, *J* = 6.9 Hz, 1H, NH), 9.31 (d, *J* = 2.2 Hz, 1H, ArH), 8.68 (d, *J* = 5.3 Hz, 1H, ArH), 8.58 (s, 1H, ArH), 7.92 (s, 1H, ArH), 7.84 (d, *J* = 5.2 Hz, 1H, ArH), 4.16–4.11 (m, 1H, NHCH), 2.64 (s, 3H, NCH_3_), 2.06–1.96 (m, 2H, cyclopentyl-H), 1.82–1.66 (m, 6H, cyclopentyl-H). ^13^C-NMR (75 MHz, Aectic-*d*_4_) δ (ppm): 163.22, 156.72, 155.84, 155.36, 149.27, 148.16, 145.80, 144.94, 137.72, 136.62, 133.83, 124.56, 121.58, 52.09, 32.74, 23.34, 20.25. HRMS (ESI): *m*/*z* [M + H]^+^ Calcd for C_19_H_21_N_6_O: 349.1771; Found: 349.1776.

##### 1-Cyclopentyl-3-(7-(3-(trifluoromethoxy)phenyl)pyrido[3,2-*d*]pyrimidin-2-yl)urea (**32c**)

White solid. Yield 53%, m.p. >250 °C. ^1^H-NMR (300 MHz, DMSO-*d*_6_) *δ* (ppm): 10.23 (s, 1H, NH), 9.51 (d, *J* = 0.8 Hz, 1H, NH), 9.38 (d, *J* = 7.2 Hz, 1H, ArH), 9.26 (d, *J* = 2.1 Hz, 1H, ArH), 8.47 (dd, *J* = 2.1, 0.8 Hz, 1H, ArH), 8.04–8.02 (m, 2H, ArH), 7.75 (t, *J* = 8.2 Hz, 1H, ArH), 7.56 (d, *J* = 8.2 Hz, 1H, ArH), 4.13–4.08 (m, 1H, NHCH), 2.01–1.91 (m, 2H, cyclopentyl-H), 1.77–1.62 (m, 6H, cyclopentyl-H). ^13^C-NMR (101 MHz, Acetic Acid-*d*_4_) *δ* (ppm): 162.67, 155.64, 155.42, 149.95 (q, *J* = 1.6 Hz), 148.69, 146.13, 140.66, 138.04, 135.46, 132.03, 131.16, 126.43, 121.78, 120.38, 119.28, 52.05, 32.74, 23.29. HRMS (ESI): *m*/*z* [M + H]^+^ Calcd for C_20_H_19_F_3_N_5_O_2_: 418.1485; Found:418.1490.

##### 1-(7-(1*H*-pyrazol-4-yl)pyrido[3,2-*d*]pyrimidin-2-yl)-3-cyclopentylurea (**32d**)

White solid. Yield 65%, m.p. >250 °C. ^1^H-NMR (300 MHz, DMSO-*d*_6_) *δ* (ppm): 13.37 (s, 1H, NH), 10.12 (s, 1H, NH), 9.42 (d, *J* = 7.0 Hz, 1H, NH), 9.38 (d, *J* = 0.8 Hz, 1H, ArH), 9.27 (d, *J* = 2.1 Hz, 1H, ArH), 8.71 (s, 1H, ArH), 8.39 (s, 1H, ArH), 8.28–8.27 (m, 1H, ArH), 4.16–4.09 (m, 1H, NHCH), 2.05–1.97 (m, 2H, cyclopentyl-H), 1.83–1.74 (m, 2H, cyclopentyl-H), 1.68–1.59 (m, 4H, cyclopentyl-H). ^13^C-NMR (75 MHz, Aectic-*d*_4_) *δ* (ppm): 161.96, 155.50, 148.01, 146.63, 141.79, 134.77, 134.47, 128.35, 128.24, 120.05, 99.90, 52.04, 32.76, 23.30. HRMS (ESI): *m*/*z* [M + H]^+^ Calcd for C_16_H_18_N_7_O: 324.1567; Found: 324.1567.

##### 1-Cyclopentyl-3-(7-(2-fluoropyridin-4-yl)pyrido[3,2-*d*]pyrimidin-2-yl)urea (**32e**)

White solid. Yield 58%, m.p. 243–244 °C. ^1^H-NMR (300 MHz, DMSO-*d*_6_) *δ* (ppm): 10.34 (s, 1H, NH), 9.58 (s, 1H, ArH), 9.39–9.36 (m, 2H, ArH, NH), 8.67 (d, *J* = 2.0 Hz, 1H, ArH), 8.50 (d, *J* = 5.2 Hz, 1H, ArH), 8.06 (d, *J* = 5.3 Hz, 1H, ArH), 7.95 (s, 1H, ArH), 4.19–4.11 (m, 1H, NHCH), 2.06–1.97 (m, 2H, cyclopentyl-H), 1.84–1.61 (m, 6H, cyclopentyl-H). ^13^C-NMR (101 MHz, Acetic Acid-*d_4_*) δ 165.53, 163.10, 155.57 (d, *J* = 39.2 Hz), 149.72 (d, *J* = 8.5 Hz), 148.43, 148.29, 148.16, 145.87, 138.05, 136.38, 133.15, 120.26 (d, *J* = 4.2 Hz), 108.28 (d, *J* = 37.5 Hz), 52.07, 32.74, 23.33. HRMS (ESI): *m*/*z* [M + H]^+^ Calcd for C_18_H_18_FN_6_O: 353.1521; Found: 353.1518.

##### 1-Benzyl-3-(7-(1-methyl-1*H*-pyrazol-4-yl)pyrido[3,2-*d*]pyrimidin-2-yl)urea (**32g**)

White solid. Yield 64%, m.p. >250 °C. ^1^H-NMR (300 MHz, DMSO-*d*_6_) *δ* (ppm): 10.27 (s, 1H, NH), 9.90 (t, *J* = 6.1 Hz, 1H, NH), 9.37 (d, *J* = 0.8 Hz, 1H, ArH), 9.19 (d, *J* = 2.1 Hz, 1H, ArH), 8.55 (s, 1H, ArH), 8.33 (dd, *J* = 2.1, 0.8 Hz, 1H, ArH), 8.22 (d, *J* = 0.8 Hz, 1H, ArH), 7.40–7.24 (m, 5H, Ph-H), 4.56 (d, *J* = 6.1 Hz, 2H, NHCH_2_), 3.93 (s, 3H, CH_3_). ^13^C-NMR (75 MHz, Aectic-*d*_4_) *δ* (ppm): 161.89, 156.14, 155.39, 147.93, 146.71, 138.54, 137.08, 134.60, 134.35, 130.10, 128.52, 127.90, 127.19, 127.17, 118.05, 43.52, 38.28. HRMS (ESI): *m*/*z* [M + H]^+^ Calcd for C_19_H_18_N_7_O: 360.1567; Found:360.1572.

##### 1-(7-(1-Methyl-1*H*-pyrazol-4-yl)pyrido[3,2-*d*]pyrimidin-2-yl)-3-phenylurea (**32h**)

White solid. Yield 63%, m.p. >250 °C. ^1^H-NMR (300 MHz, DMSO-*d*_6_) *δ* (ppm): 11.83 (s, 1H, NH), 10.57 (s, 1H, NH), 9.44 (d, *J* = 0.8 Hz, 1H, ArH), 9.26 (d, J = 2.1 Hz, 1H, ArH), 8.65 (s, 1H, ArH), 8.50 (dd, *J* = 2.1, 0.9 Hz, 1H, ArH), 8.37 (d, *J* = 0.8 Hz, 1H, ArH), 7.81–7.78 (m, 2H, Ph-H), 7.43–7.38 (m, 2H, Ph-H), 7.15–7.10 (m, 1H, Ph-H), 3.96 (s, 3H, CH_3_). ^13^C-NMR (75 MHz, Aectic-*d*_4_) *δ* (ppm): 172.87, 162.13, 158.12, 143.34, 134.80, 131.85, 130.31, 128.92, 128.64, 124.36, 123.00, 120.22, 120.05, 118.07, 114.16, 64.04. HRMS (ESI): *m*/*z* [M + H]^+^ Calcd for C_18_H_16_N_7_O: 346.1411; Found: 346.1404.

##### 1-Ethyl-3-(7-(1-methyl-1*H*-pyrazol-4-yl)pyrido[3,2-*d*]pyrimidin-2-yl)urea (**32i**)

White solid. Yield 82%, m.p. >250 °C. ^1^H-NMR (300 MHz, DMSO-*d*_6_) *δ* (ppm): 10.10 (s, 1H, NH), 9.41 (t, *J* = 5.6 Hz, 1H, NH), 9.35 (d, *J* = 0.8 Hz, 1H, ArH), 9.19 (d, *J* = 2.1 Hz, 1H, ArH), 8.58 (s, 1H, ArH), 8.36 (dd, *J* = 2.1, 0.8 Hz, 1H, ArH), 8.26 (d, *J* = 0.7 Hz, 1H, ArH), 3.94 (s, 3H, CH_3_), 3.34–3.33 (m, 2H, CH_2_CH_3_), 1.22 (t, *J* = 7.2 Hz, 3H, CH_2_CH_3_). ^13^C-NMR (75 MHz, Aectic-*d*_4_) *δ* (ppm): 146.13, 144.52, 141.58, 139.66, 135.42, 135.26, 132.75, 129.80, 126.87, 120.95, 118.00, 61.99, 59.04, 50.19. HRMS (ESI): *m*/*z* [M + H]^+^ Calcd for C_14_H_16_N_7_O: 298.1411; Found: 298.1407.

##### 1-(7-(1-Methyl-1*H*-pyrazol-4-yl)pyrido[3,2-*d*]pyrimidin-2-yl)-3-(2-morpholinoethyl)urea (**32j**)

White solid. Yield 46%, m.p. 222–224 °C. ^1^H-NMR (300 MHz, CDCl_3_) *δ* (ppm): 9.61 (s, 1H, NH), 9.38 (s, 1H, NH), 9.06 (d, *J* = 2.0 Hz, 1H, ArH), 8.18 (s, 1H, ArH), 8.02 (s, 1H, ArH), 7.95 (s, 1H, ArH), 7.87 (s, 1H, ArH), 4.08 (s, 3H, CH3), 3.98–3.85 (m, 4H, O(CH_2_)_2_), 3.78–3.65 (m, 2H, CH_2_NH), 2.85–2.62 (m, 6H, N(CH_2_)_3_). ^13^C-NMR (75 MHz, Aectic-*d*_4_) *δ* (ppm): 156.57, 155.07, 148.19, 146.73, 137.24, 134.71, 134.39, 130.40, 128.17, 120.06, 118.10, 63.68, 57.13, 52.33, 38.45, 34.50. HRMS (ESI): *m*/*z* [M + H]^+^ Calcd for C_18_H_23_N_8_O_6_: 383.1938; Found:383.1939.

##### 1-Cyclopentyl-3-(7-(2-methylpyridin-4-yl)pyrido[3,2-*d*]pyrimidin-2-yl)urea (**32k**)

White solid. Yield 50%, m.p. >250 °C. ^1^H-NMR (300 MHz, DMSO-*d*_6_) *δ* (ppm): 12.35 (s, 1H, NH), 9.40 (s, 1H, ArH), 9.19 (d, *J* = 2.0 Hz, 1H, ArH), 8.64 (s, 1H, ArH), 8.32 (d, *J* = 2.9 Hz, 2H, ArH), 3.94 (s, 3H, NCH_3_), 2.66 (s, 3H, CCH_3_). ^13^C-NMR (75 MHz, Aectic-*d*_4_) *δ* (ppm): 161.34, 161.23, 152.63, 147.02, 146.72, 136.94, 134.14, 133.76, 130.03, 126.80, 120.06, 117.64, 38.19, 13.57. HRMS (ESI): *m*/*z* [M + H]^+^ Calcd for C_14_H_13_N_8_S: 325.0978; Found:325.0984.

##### 1-Cyclopentyl-3-(7-(1-(2-morpholinoethyl)-1*H*-pyrazol-4-yl)pyrido[3,2-*d*]pyrimidin-2-yl)urea (**32l**)

White solid. Yield 51%, m.p.129–131 °C. ^1^H-NMR (300 MHz, CDCl_3_) *δ* (ppm): 9.34–9.30 (m, 2H, NHCONH), 9.03–9.02 (m, 1H, ArH), 8.08 (s, 1H, ArH), 8.02 (s, 1H, ArH), 7.94 (d, *J* = 2.0 Hz, 1H, ArH), 7.73 (s, 1H, ArH), 4.52–4.32 (m, 3H, NHCH, NCH_2_), 3.86–3.70 (m, 4H, CH_2_OCH_2_), 3.12–2.94 (m, 2H, NCH_2_), 2.63–2.57 (m, 4H, N(CH_2_)_2_), 2.17–2.01 (m, 2H, cyclopentyl-H), 1.88–1.75 (m, 6H, cyclopentyl-H). ^13^C-NMR (75 MHz, DMSO-*d*_6_) *δ* (ppm): 163.38, 156.20, 153.82, 148.46, 146.54, 135.36, 134.71, 130.23, 126.39, 120.52, 117.98, 66.61, 60.23, 53.55, 51.71, 33.26, 23.71, 14.56. HRMS (ESI): *m*/*z* [M + H]^+^ Calcd for C_22_H_29_N_8_O_2_: 437.2408; Found:437.2410.

##### 1-Cyclopentyl-3-(7-(1-(2-(dimethylamino)ethyl)-1H-pyrazol-4-yl)pyrido[3,2-d]pyrimidin-2-yl) urea (**32m**)

White solid. Yield 42%, m.p.190–192 °C. ^1^H-NMR (300 MHz, CDCl_3_) *δ* (ppm): 9.39–9.27 (m, 2H, NHCONH), 9.03 (d, *J* = 2.1 Hz, 1H, ArH), 8.06 (s, 1H, ArH), 8.02 (s, 1H, ArH), 7.95–7.94 (m, 1H, ArH), 7.80 (s, 1H, ArH), 4.42–4.30 (m, 3H, NHCH, NCH_2_), 2.92 (t, *J* = 6.3 Hz, 2H, NCH_2_CH_2_), 2.37 (s, 6H, (CH_3_)_2_), 2.15–2.10 (m, 2H, cyclopentyl-H), 1.87–1.83 (m, 2H, cyclopentyl-H), 1.77–1.68 (m, 4H, cyclopentyl-H). ^13^C-NMR (75 MHz, DMSO-*d*_6_) *δ* (ppm): 163.39, 156.21, 153.83, 148.48, 146.56, 138.08, 135.34, 134.74, 130.19, 126.34, 117.90, 58.85, 51.72, 50.28, 45.56, 33.24, 23.71. HRMS (ESI): *m*/*z* [M + H]^+^ Calcd for C_20_H_27_N_8_O_2_: 395.2302; Found:395.2309.

#### 3.1.20. *tert*-Butyl 4-(2-(3-cyclopentylureido)pyrido[3,2-*d*]pyrimidin-7-yl)-3,6-dihydropyridine-1(2*H*)-carboxylate (**31**)

This procedure is the same as the general synthesis of **13a** and **13b**.

White solid. Yield 42%, m.p.190–192 °C. ^1^H-NMR (300 MHz, DMSO-*d*_6_) *δ* (ppm): 10.15 (s, 1H, NH), 9.40 (s, 1H, ArH), 9.38 (d, *J* = 7.2 Hz, 1H, NH), 9.10 (d, *J* = 2.1 Hz, 1H, ArH), 8.03 (s, 1H, ArH), 6.73–6.69 (m, 1H, CHC), 4.16–4.11 (m, 3H, NHCH, NCH2), 3.64 (t, *J* = 5.7 Hz, 2H, NCH2), 2.69–2.64 (m, 2H, NCH_2_CH_2_), 2.00–1.94 (m, 2H, cyclopentyl-H), 1.81–1.75 (m, 2H, cyclopentyl-H), 1.68–1.61 (m, 4H, cyclopentyl-H), 1.47 (s, 9H, C(CH_3_)_3_).

#### 3.1.21. 1-Cyclopentyl-3-(7-(1,2,3,6-tetrahydropyridin-4-yl)pyrido[3,2-*d*]pyrimidin-2-yl)urea hydrochloride (**32f**)

To a solution of Boc-protected compound (0.16 mmol) in EA (2 mL), HCl (EA) (2 mL) was added at 0 °C and stirred at r.t for 2 h. After completion (monitored by TLC), the mixture was concentrated under reduced pressure and the residue was purified by refining (*V*_DCM_:*V*_MeOH_ = 20:1) to give the product **32f**.

White solid. Yield 83%, m.p.172–174 °C. ^1^H-NMR (300 MHz, DMSO-*d*_6_) *δ* (ppm): 10.15 (s, 1H, NH), 9.42 (s, 1H, ArH), 9.38 (d, *J* = 7.2 Hz, 1H, NH), 9.10 (d, *J* = 2.1 Hz, 1H, ArH), 8.03 (s, 1H, ArH), 6.71 (s, 1H, CHC), 4.17–4.12 (m, 3H, NHCH_2_, NHCH), 3.64 (t, *J* = 5.7 Hz, 2H, NHCH_2_), 2.69–2.64 (m, 2H, CH_2_C), 2.00–1.93 (m, 2H, cyclopentyl-H), 1.82–1.74 (m, 2H, cyclopentyl-H), 1.67–1.57 (m, 4H, cyclopentyl-H). ^13^C-NMR (75 MHz, Acetic-*d*_4_) *δ* (ppm): 162.27, 155.63, 147.35, 140.44, 135.20, 131.79, 129.86, 122.46, 52.05, 42.47, 40.85, 39.02, 38.98, 32.74, 23.29, 22.99. HRMS (ESI): *m*/*z* [M + H]^+^. Calcd for C_18_H_23_N_6_O: 339.1928; Found: 339.1923.

### 3.2. Enzymatic Inhibition Assays

The 50 µL reaction mixture contains 40 mM Tris-HCl, pH 7.4, 10 µM ATP, 10 mM MgCl_2_, 0.1 mg/mL BSA, 1 mM DTT, 0.2 ug/mL ERK 2, ERK 1, PI3Kα, PI3Kβ, PI3Kγ or PI3Kδ and 100 uM lipid substrate. All compounds were diluted in 10% DMSO and tested in 10-dose with 3-fold serial dilution starting at a concentration of 1 μM. Of the dilution, 5 µL was added to a 50 µL reaction so that the final concentration of DMSO is 1% in all of reactions. After all of the reaction mixtures were conducted at 30 °C for 40 min, the assay was performed using ADP-Glo Plus luminescence kinase assay kit. The luminescence signal and the intensity of the luminescence signal value were detected using a multi-well spectrophotometer (the MD-SpectraMax M5 multifunctional microplate reader). It was directly proportional to the inhibition of enzyme activity. The experimental results were converted into active percentages, a dose-response curve is drawn, and the IC_50_ value of inhibition was calculated using GRAPHPAD PRISM 5 nonlinear regression.

### 3.3. Anti-Proliferation Assay

Human cancer cell lines HCT116 and HEC1B were cultured in media with 10% FBS (GIBCO, Invitrogen Corporation, NY, USA) at 37 °C in a 5% (*v*/*v*) CO_2_ humidified incubator. The logarithmic growth phase cells were seeded in a 96-well plate with a density of 1 × 10^5^ cells/mL, cultured at 37 °C, 5% CO_2_. Until the cells were 90% confluent, the medium containing compound was added to incubate for 72 h, and then the cell viability was tested by MTT analysis. Taking the drug concentration as the abscissa and the percentage of proliferation inhibitory activity corresponding to each concentration as the ordinate, using Graphpad Prism 5 to do nonlinear regression, the IC_50_ value of each compound was calculated.

### 3.4. In Vitro Pharmacokinetic Study

The pharmacokinetic experiment was conducted by using human liver microsome (purchased from Research Institute for Liver Diseases (Shanghai) Co., Ltd., Shanghai, China). The reaction mixture contained 100 μL PBS, 40 μL liver microsome and 20 μL dilution of compound **32d**. After the reaction mixtures were preconducted at 37 °C for 5 min, 40 μL NADPH solution was added to start pharmacokinetic reaction, and samples were collected at different time points (5 min, 10 min, 15 min, 30 min and 45 min). Then, 100 μL of ethyl acetate was added to stop the reaction. The whole samples were added in 10 μL of an internal standard (Phenacetin) to shake for 10 min and centrifuge at 10,000 rpm for 10 min and then taken out 300 μL of supernatant.for LC/MS/MS analysis. WinNolin 8.2 software was used to calculate pharmacokinetic parameters, and GraphPad Prism 8.0 was used to draw concentration-time curves.

### 3.5. In Vivo Pharmacokinetic Study

The pharmacokinetic experiment was conducted by using 180–220 g male Sprague Dawley rats (purchased from Weitong Lihua Experimental Animal Co., Ltd., Beijing, China). The experimental animals were raised in a well-ventilated, air-conditioned standard animal room where the temperature was maintained at 20–25 °C, the humidity was maintained at 40–70%, and the light and dark were rotated for 12 h each. After about 5 days of normal feeding, rats with good physical signs can enter this experiment after veterinary inspection. All rats were fasted for 12 h before the start of the animal experiment. The **32d** was dissolved in a mixture of 40% DMSO + 40% PEG400 + 20% 5% glucose injection at a concentration of 1 mg/mL (1 mg/kg). The **32d** was administered to the mice by p.o. (0.2 mg/mL, 10 mg/kg), and blood samples (about 0.25 mL) were collected from the posterior orbital venous plexus after different time points (i.v.: 2 min, 15 min, 30 min, 1 h, 2 h, 4 h, 6 h, 8 h, and 12 h; po: 5 min, 15 min, 30 min, 1 h, 2 h, 4 h, 6 h, 8 h, 12 h, and 24 h) after dosing, with three mice for each time point. The whole plasma samples were kept frozen at −20 °C until LC/MS/MS analysis after centrifugation. WinNolin 8.2 software was used to calculate pharmacokinetic parameters, and GraphPad Prism 8.0 was used to draw plasma concentration-time curves.

### 3.6. In Vivo Antitumor Activity Evalution

Male five-week-old Balb/c-nude mice (18–20 g) were injected subcutaneously with HCT-116 cells (5 × 10^7^/mL cells suspended in Matrigel Matrix). When the tumor reached a volume of approximately 100 mm^3^, the mice were randomly assigned to five groups (six mice for each group) and treated with vehicle (5% (*v*/*v*) DMSO), **32d** (5 mg/kg), BVD-523 (5 mg/kg), GDC-0980 (1 mg/kg) and BVD-523 + GDC-0980 (2.5 mg/kg + 0.5 mg/kg). All mice were administered by intraperitoneal q.d. The tumor volumes were measured using electronic digital calipers every 3 days and were calculated as 1/2 length × width × width.

### 3.7. Molecular Docking

The X-ray crystal structures of ERK2 (PDB ID: 5KE0) and PI3Kα (PDB ID: 4JPS) were downloaded from the Protein Data Bank. The protein structures were prepared using Protein Preparation Wizard of the Schrodinger Suite to ensure that all the water molecules and solvent molecules of downloaded X-ray structure were removed. LigPrep was used to minimize the ionized conformer and tautomeric states of the small molecules. Finally, after the receptor grid was generated, the prepared ligands can be docked into the receptor protein through the Glide implemented in Schrodinger 2013. The best pose, with lowest energy conformations and appropriate hydrogen-bond geometries, was output.

## 4. Conclusions

In summary, a novel series of pyrido[3,2-*d*]pyrimidine derivatives as ERK and PI3K dual inhibitors were designed, synthesized and identified. Some of these compounds have excellent ERK and PI3K inhibitory activity. Preliminary SAR investigation led to the identification of **32d**, a potent and highly efficacious ERK and PI3K dual inhibitor. Compound **32d** exhibited moderate ERK and PI3K inhibitory activities and anti-proliferation potencies. Although **32d** only possessed acceptable pharmacokinetic profiles with a moderate half-life (t_1/2_ = 2.32 h) of intravenous administration in SD rats, it showed considerable activity in vivo antitumor efficacy in a HCT-116 xenograft model without causing observable toxic effects. All the results indicated that **32d** provided a promising basis for further optimization towards dual ERK/PI3K inhibitors. In addition, further studies will be carried out in the near future.

## Figures and Tables

**Table 1:**
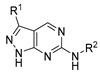
In vitro ERK 2 and PI3Kα inhibitory activities of compounds **16a**~**16d**.

Entry	R^1^	R^2^	ERK2 Inhibition% (1 μM)	PI3Kα Inhibition% (1 μM)
**16a**	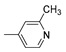	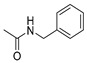	98.3	8.7
**16b**	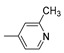	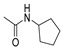	84.8	27.4
**16c**	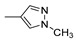	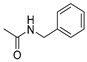	97.2	1.8
**16d**	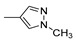	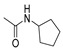	79.5	4.0
**BVD-525**			97.7	ND
**BYL-719**			ND	96.7

ND = not determined.

**Table 2:**
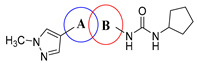
In vitro ERK 2 and PI3Kα inhibitory activities of compounds **24** and **32a**.

Entry	AB	ERK2 Inhibition% (1 μM)	PI3Kα Inhibition% (1 μM)
**24**	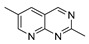	1.8	15.1
**32a**	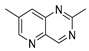	89.9	85.0

**Table 3:**
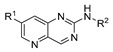
In vitro ERK 2 and PI3Kα inhibitory activities of compounds **32b**~**32m**.

Entry	R^1^	R^2^	ERK2 Inhibition% (100 nM)	PI3Kα Inhibition% (100 nM)
**32b**	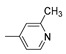	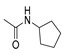	53.7	10.5
**32c**	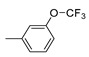	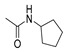	1.6	−11.3
**32d**	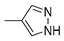	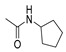	49.3	51.4
**32e**	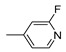	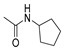	35.4	7.0
**32f**	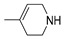	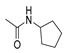	0.4	−8.8
**32g**	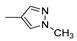	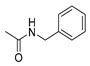	55.2	32.4
**32h**	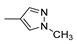	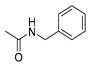	17.2	24.9
**32i**	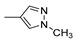	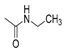	22.1	33.4
**32j**	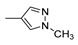	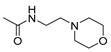	6.2	15.1
**32k**	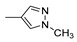	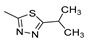	66.5	15.3
**32l**	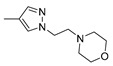	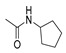	21.4	63.8
**32m**	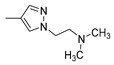	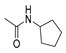	8.4	41.0
**BVD-525**			97.7	ND
**BYL-719**			ND	98.2

ND = not determined.

**Table 4:**
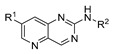
In vitro ERK 2 and PI3Kα IC_50_ of compounds **32a**, **32d** and **32g**.

Entry	R^1^	R^2^	ERK2 IC_50_ (nM)	PI3Kα IC_50_ (nM)
**32a**	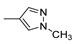	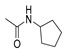	89	38
**32d**	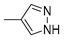	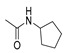	73	59
**32g**	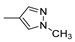	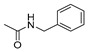	83	824

**Table 5:** In vitro ERK 1, PI3Kβ, PI3Kγ and PI3Kδ inhibitory activities of compound **32d**.

Entry	ERK1 Inhibition% (100 nM)	PI3Kβ Inhibition% (100 nM)	PI3Kγ Inhibition% (100 nM)	PI3Kδ Inhibition% (100 nM)
**32a**	39.3%	13.9%	43.2%	11.1%

**Table 6:** In vitro anti-proliferation activities of selected compounds.

Entry	IC_50_ (μM)	
	HEC1B	HCT116
**32a**	1.848	1.945
**32d**	1.492	0.7255
**32g**	2.272	10
**32l**	3.63	1.76
**BVD-523**	18.02	0.3209
**GDC-0980**	0.2295	1.065

**Table 7:** Oxidation metabolism parameters of **32d** in human liver microsomes (*n* = 3).

Parameters	Value
K (min^−1^)	0.004
t_1/2_ (min)	173.25
P_m_ (mg/mL)	0.25
Cl_int_ (mL/min/mg)	0.016

**Table 8:** PK Parameters of **32d** in SD Rats (*n* = 3)

Comp. i.v./p.o.	Dose mg/kg	CL mL/kg/ min	Vz mL/kg	AUC_0–t_ ng·h/mL	AUC_0–∞_ ng·h/mL	MRT_0–t_ h	t_1/2_ h	C_max_ ng/mL	F%
**32d** (i.v.)	1	44.8	9124	368.0	379.0	0.99	2.32	1306.7	--
**32d** (p.o.)	10	727.9	244740	191.5	355.1	2.93	2.65	128.8	9.37

## Supplementary Materials

The following are available online, Figure S1: Dose-inhibition response curves of compounds **32a**, **32d**, **32g**, 32l, BVD-523 and GDC-0980.
