# Supplementary material for: Discovery of Novel Dual Extracellular Regulated Protein Kinases (ERK) and Phosphoinositide 3-Kinase (PI3K) Inhibitors as a Promising Strategy for Cancer Therapy
Source: Molecules. 2020 Dec 3;25(23):5693. doi: 10.3390/molecules25235693 (PMC7730961; doi:10.3390/molecules25235693)
Supplement: Supplementary file 1 [file molecules-25-05693-s001.pdf]

Supplementary Materials

Discovery of Novel Dual Extracellular Regulated Protein Kinases (ERK) and Phosphoinositide 3-Kinase (PI3K) Inhibitors as A Promising Strategy for Cancer Therapy

Lingzhi Zhang [a,1], Qiurong Ju [a,1], Jinjin Sun [a], Lei Huang [1], Shiqi Wu [a], Shuping Wang [a], Yin Li [a], Zhe Guan [a], Qihua Zhu [a, b,] ** and Yungen Xu [a, b,] *

[a] State Key Laboratory of Natural Medicines, China Pharmaceutical University, Nanjing, 210009, China; zhanglingzhicpu@163.com (L.Z.); jqr474678912@163.com (Q.J.); fas15850601108@163.com (J.S.); 18361073522@163.com (L.H.); wushiqicpu1@163.com (S.W.) (Shiqi Wu); wangsp16@126.com (S.W.) (Shuping Wang); linny_cpu@126.com (Y.L.); guanz2008@163.com (Z.G)

[b] Jiangsu Key Laboratory of Drug Design and Optimization, Department of Medicinal Chemistry, China Pharmaceutical University, Nanjing 210009, China

* Correspondence: zhuqihua@vip.126.com (Q.Z.); xyg@cpu.edu.cn (Y.X.); Tel.: +86-025-86185303 (Y.X.)

(Supporting Information)

^{1}H and ^{13}C-NMR spectra of compounds **16a~16d**--S2–S5

^{1}H and ^{13}C-NMR spectra of compounds **24** --S6

^{1}H and ^{13}C-NMR spectra of compounds **32a~32m**--S7–S19

Dose-inhibition response curves of compounds **32a**, **32d**, **32g**, **32l,** BVD-523 **and** GDC-0980---S20–S21

Compound **16a**

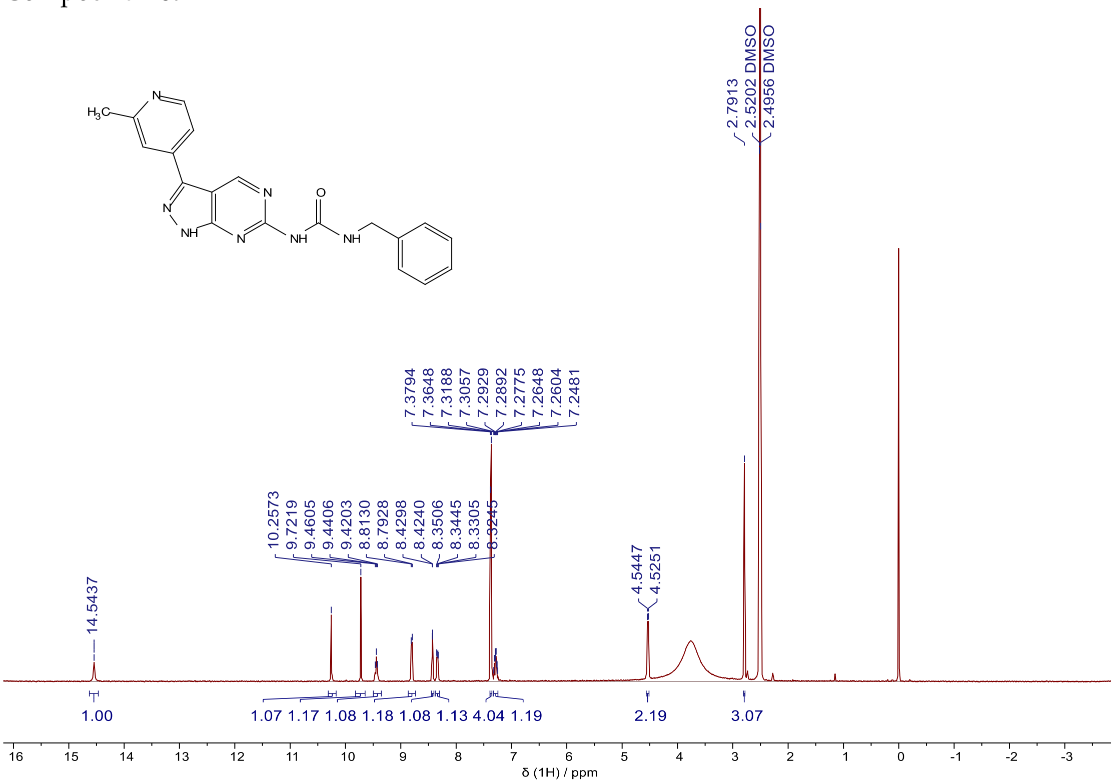

^{1}H-NMR spectra of compound **16a**

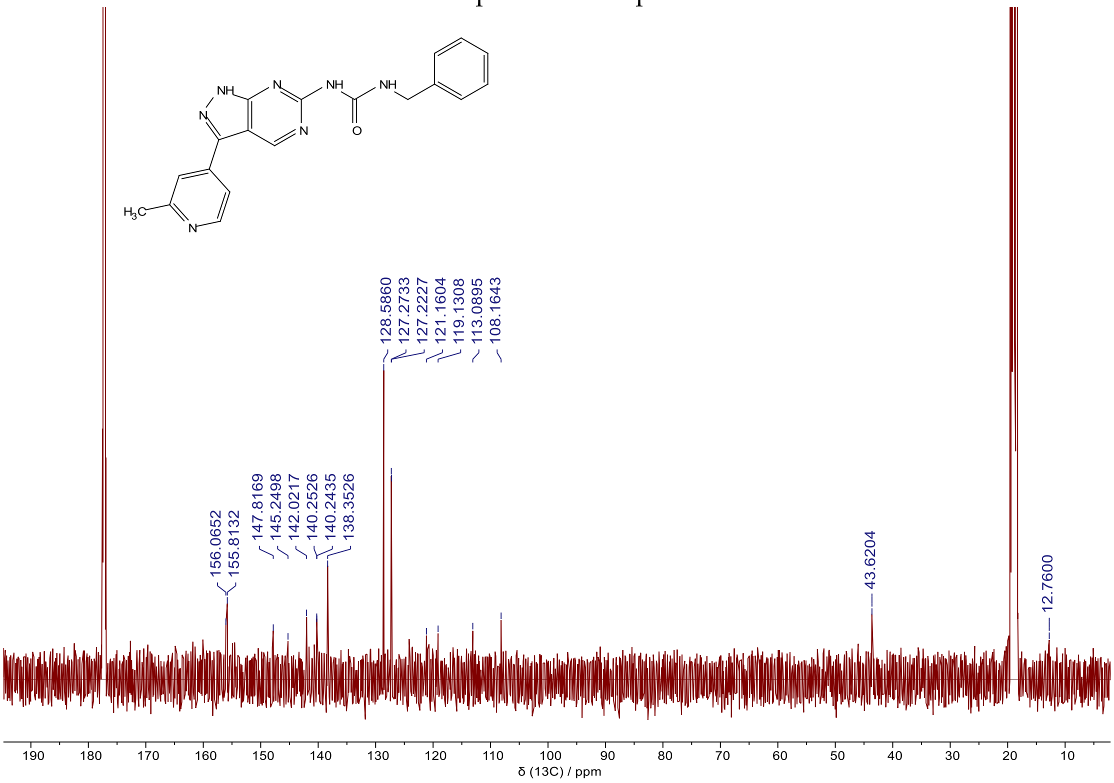

^{1}C-NMR spectra of compound **16a**

Compound **16b**

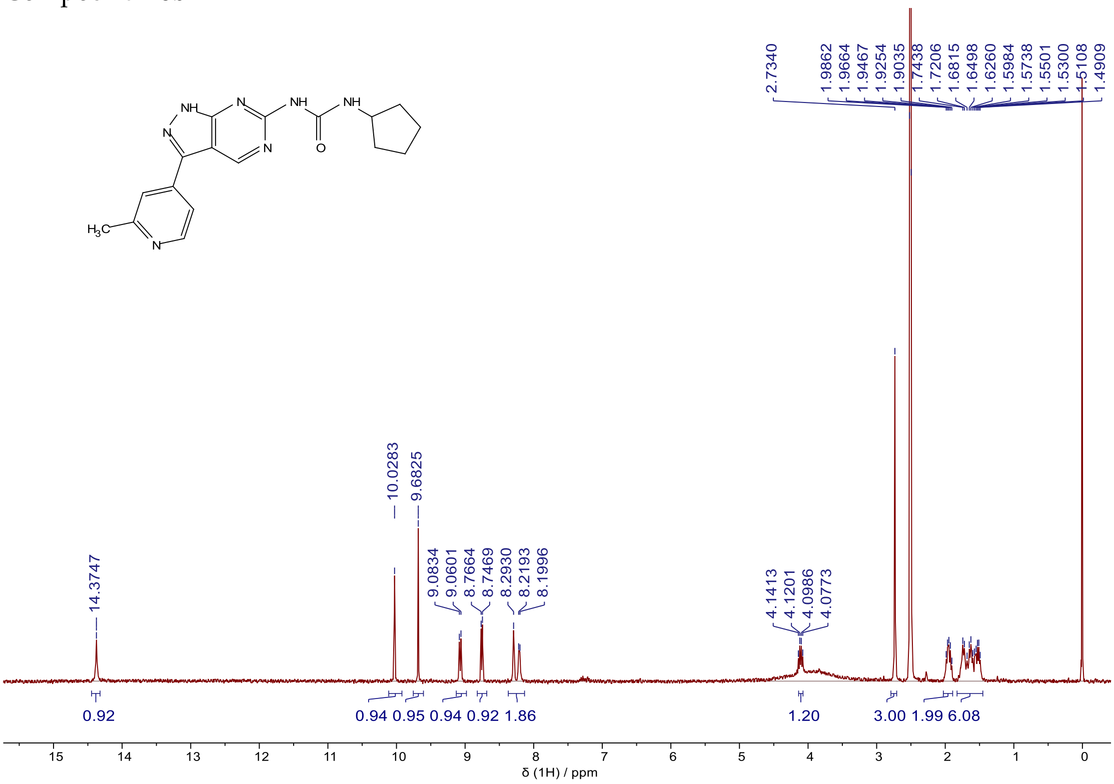

^{1}H-NMR spectra of compound **16b**

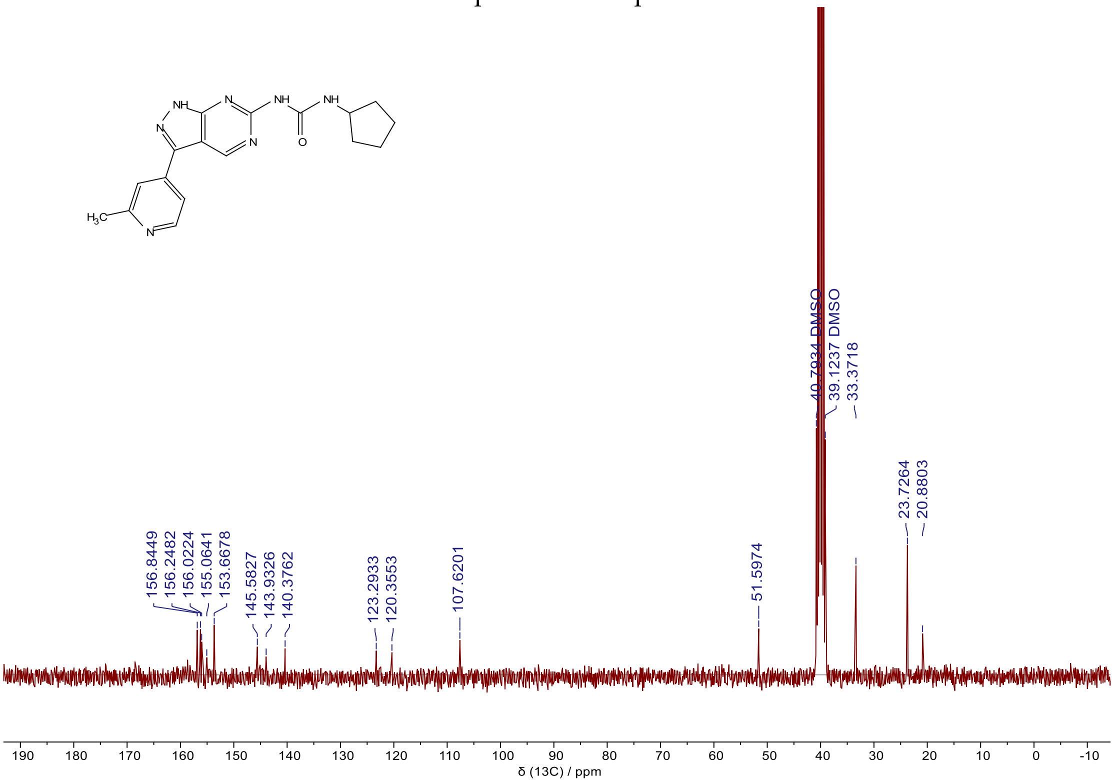

^{13}C-NMR spectra of compound **16b**

Compound **16c**

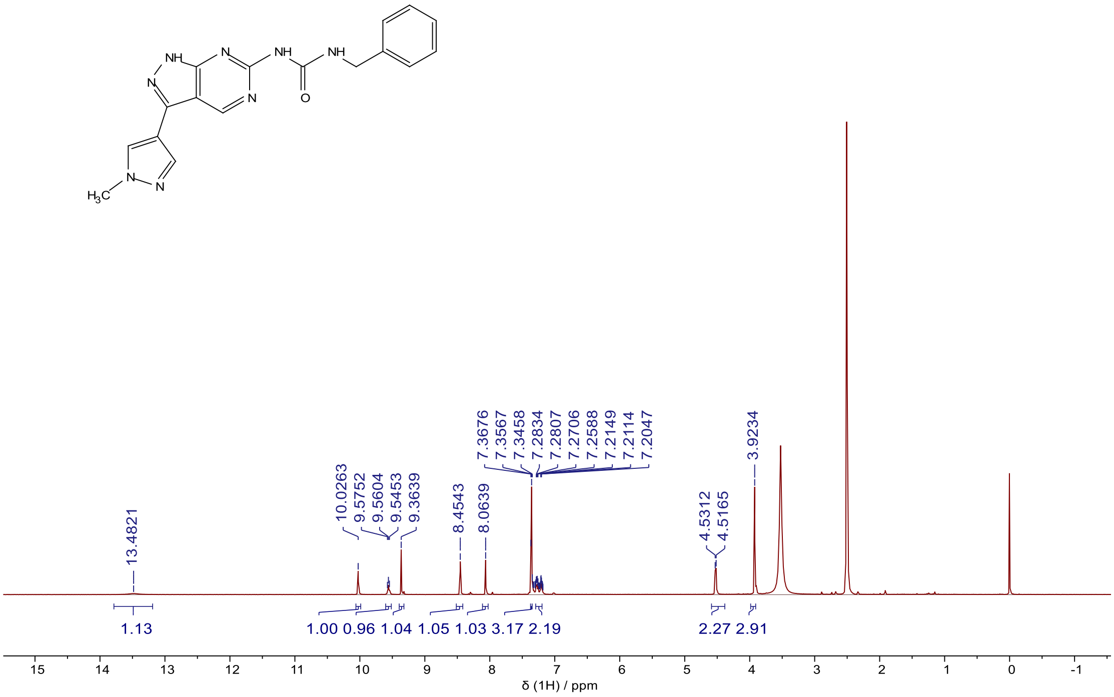

^{1}H-NMR spectra of compound **16c**

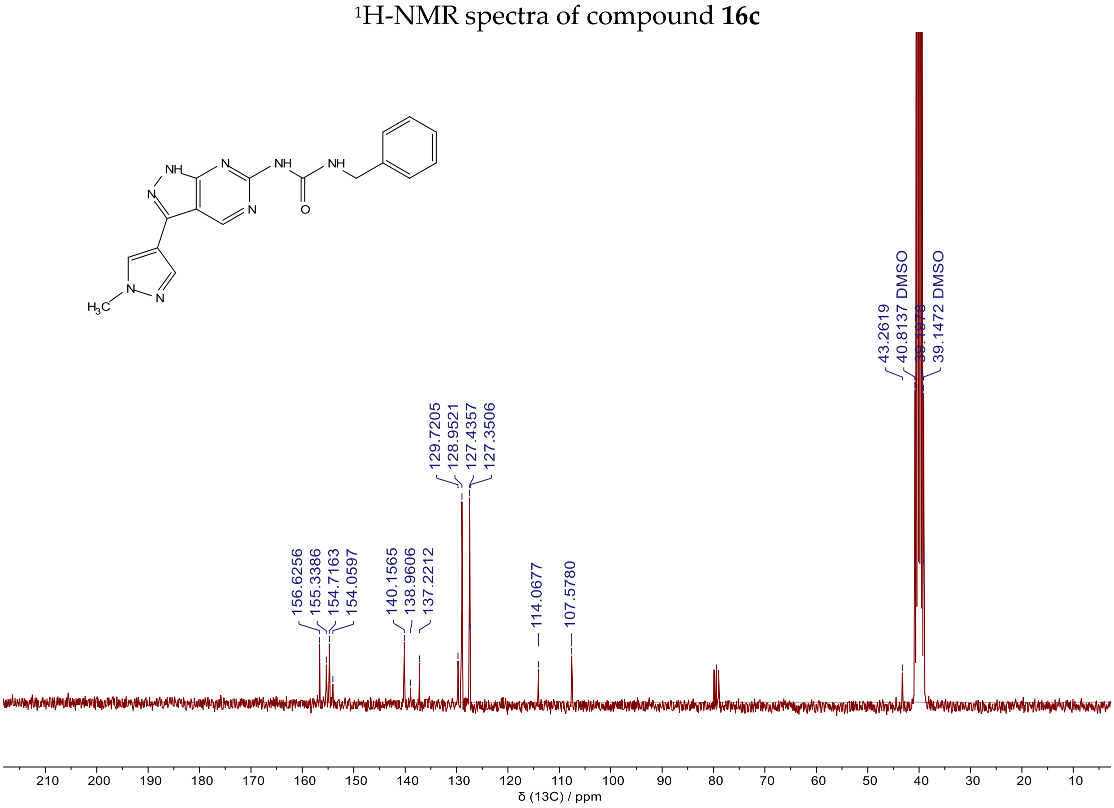

^{13}C-NMR spectra of compound **16c**

Compound **16d**

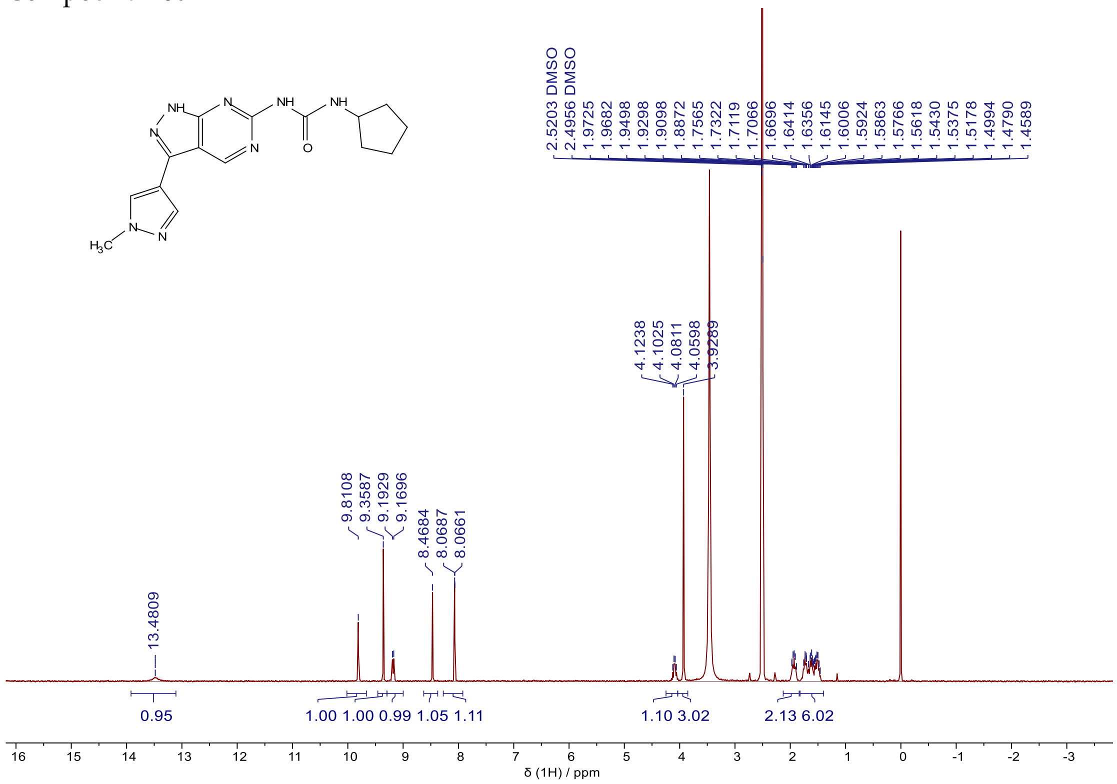

^{1}H-NMR spectra of compound **16d**

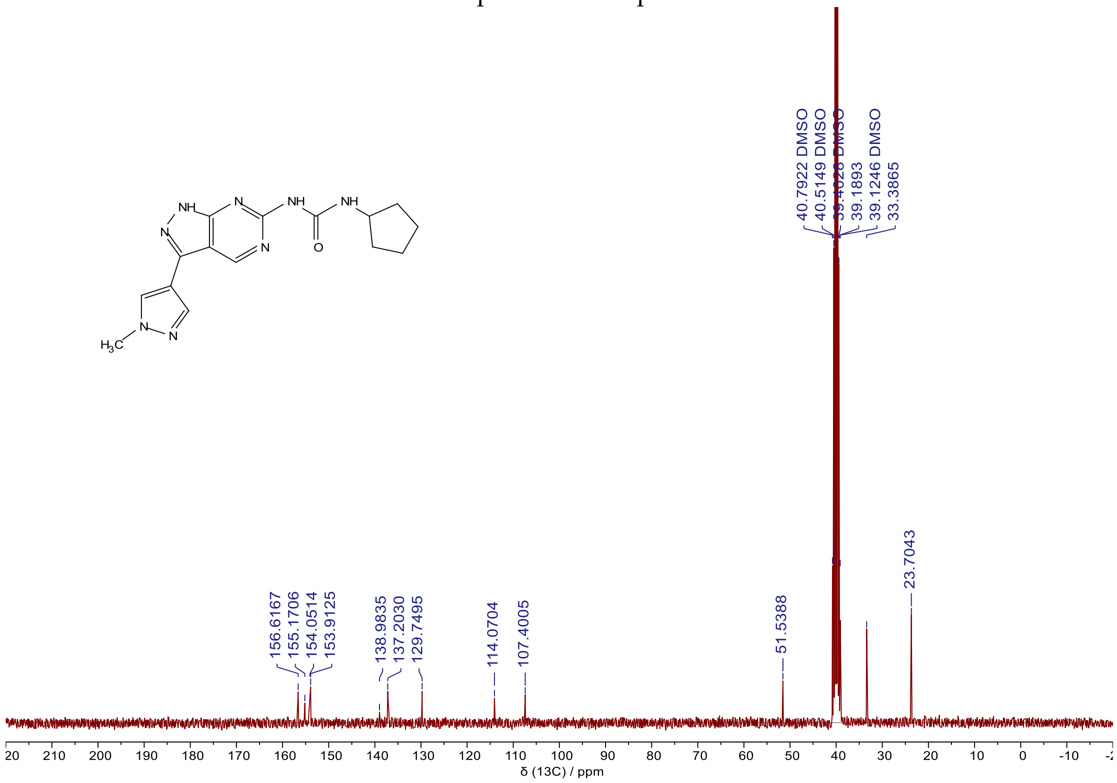

^{13}C-NMR spectra of compound **16d**

Compound **24**

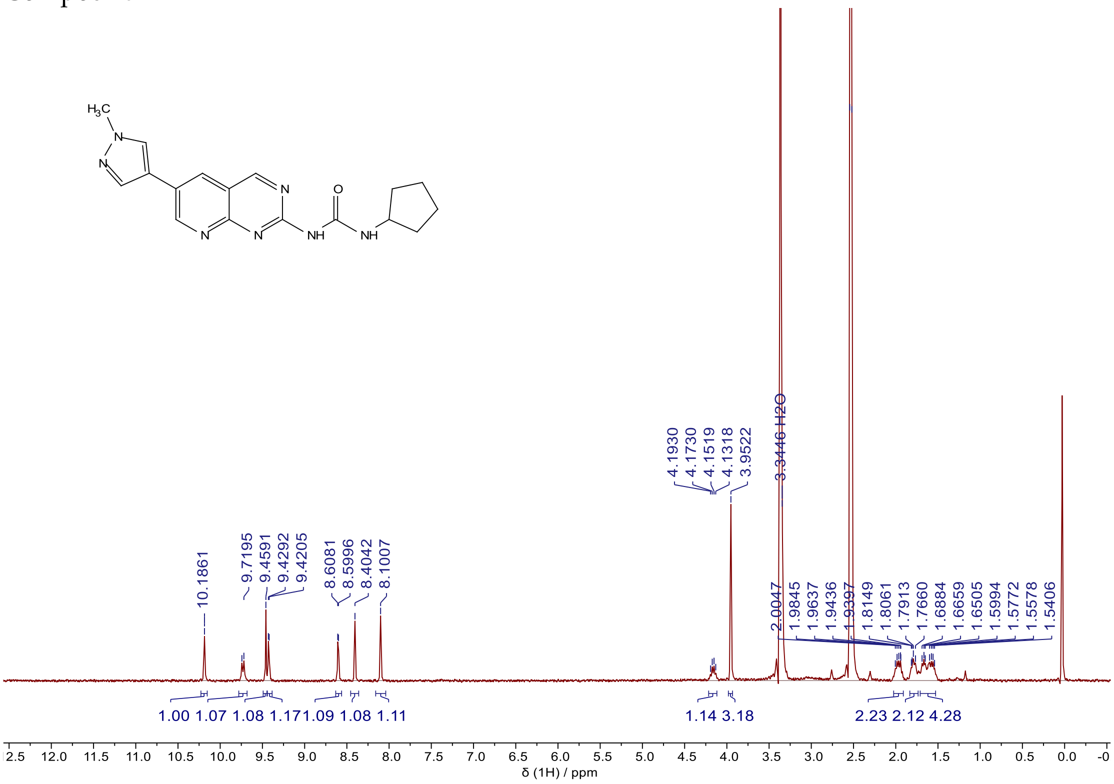

^{1}H-NMR spectra of compound **24**

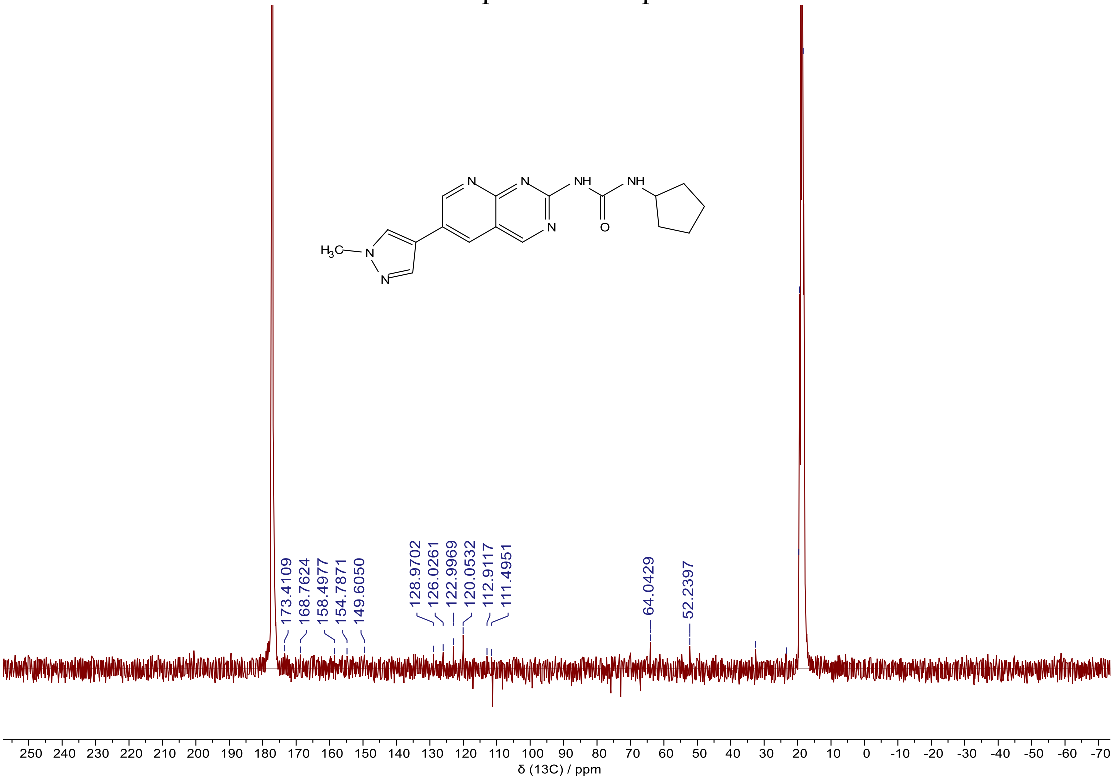

^{13}C-NMR spectra of compound **24**

Compound **32a**

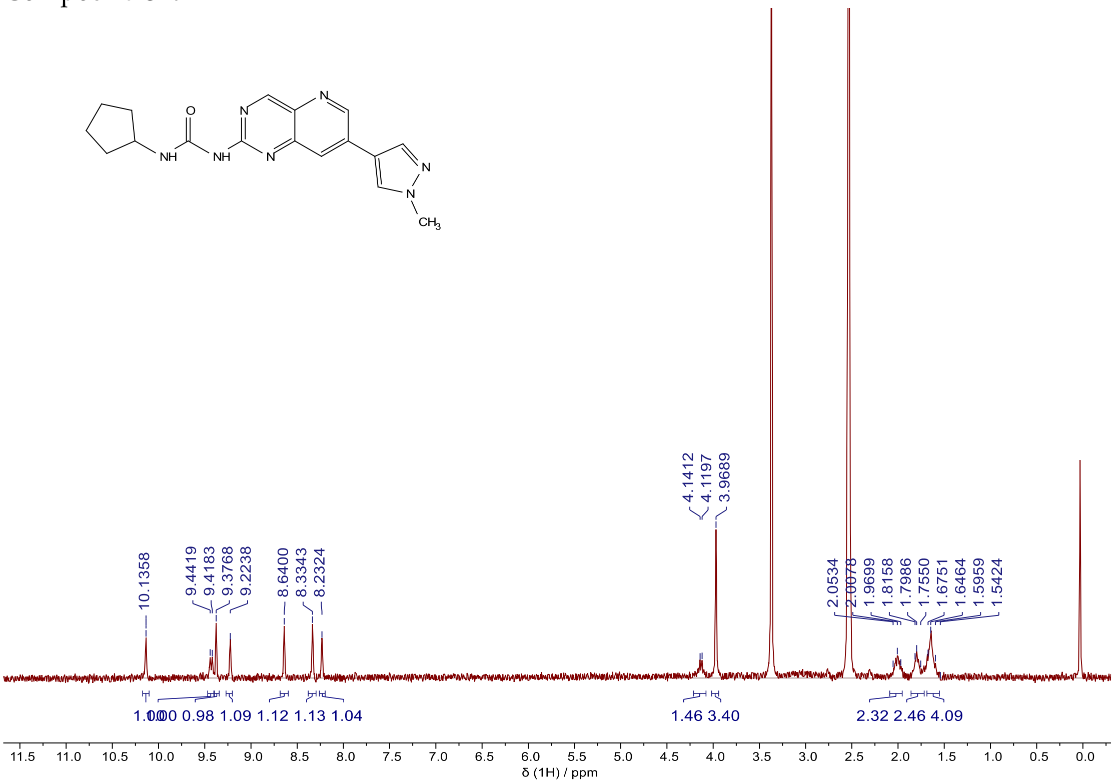

^{1}H-NMR spectra of compound **32a**

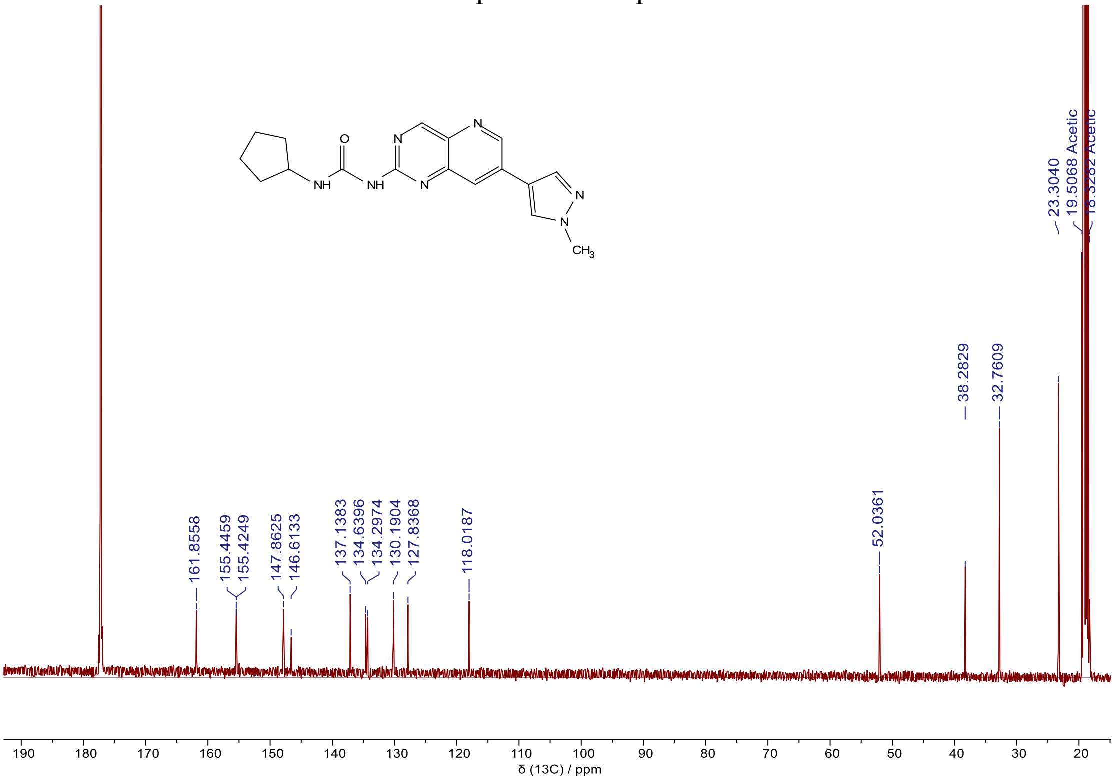

^{13}C-NMR spectra of compound **32a**

Compound **32b**

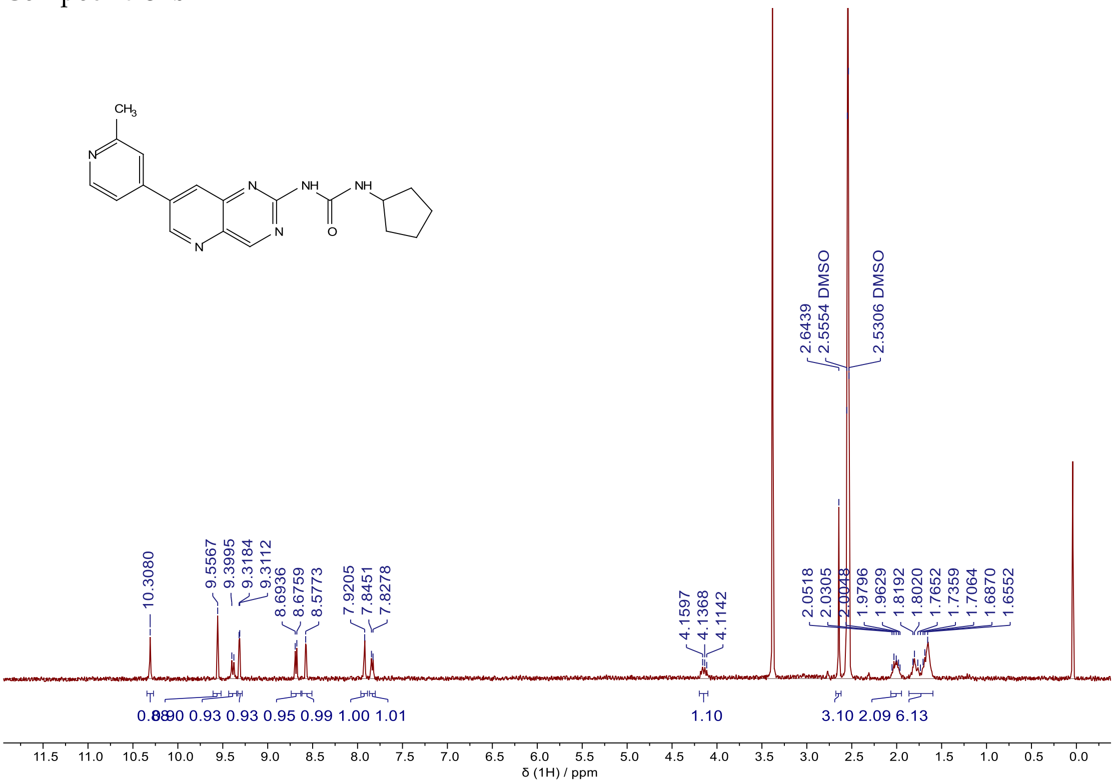

^{1}H-NMR spectra of compound **32b**

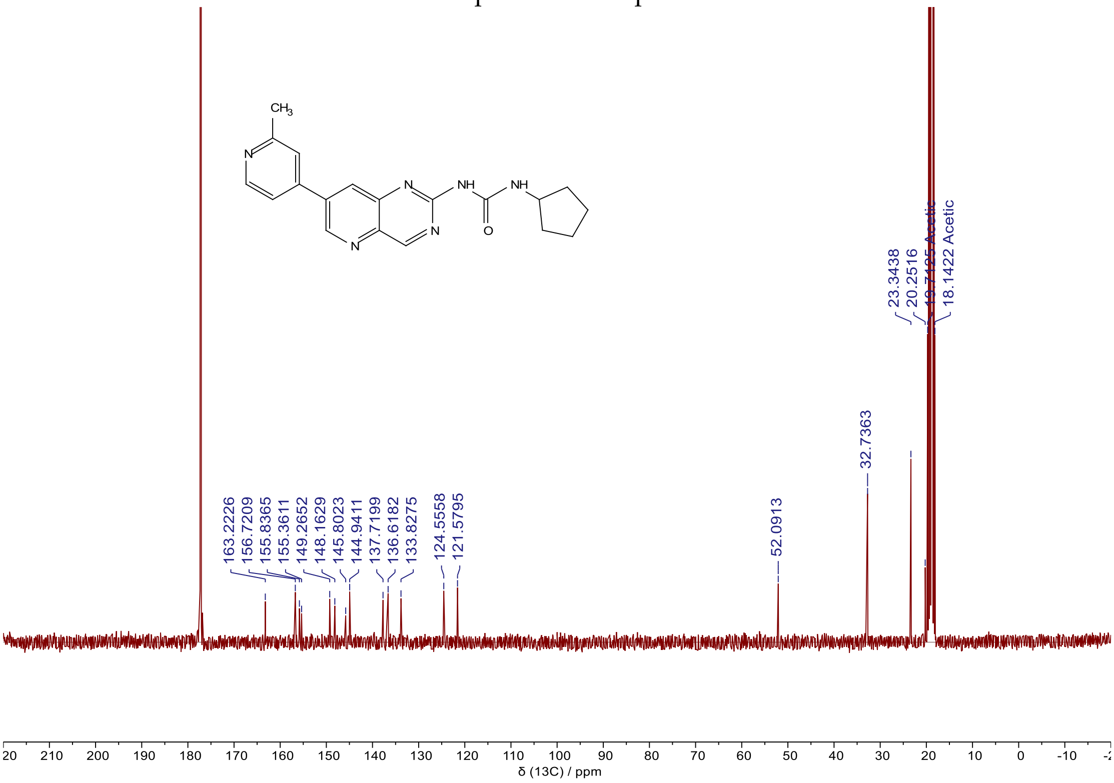

^{13}C-NMR spectra of compound **32b**

Compound **32c**

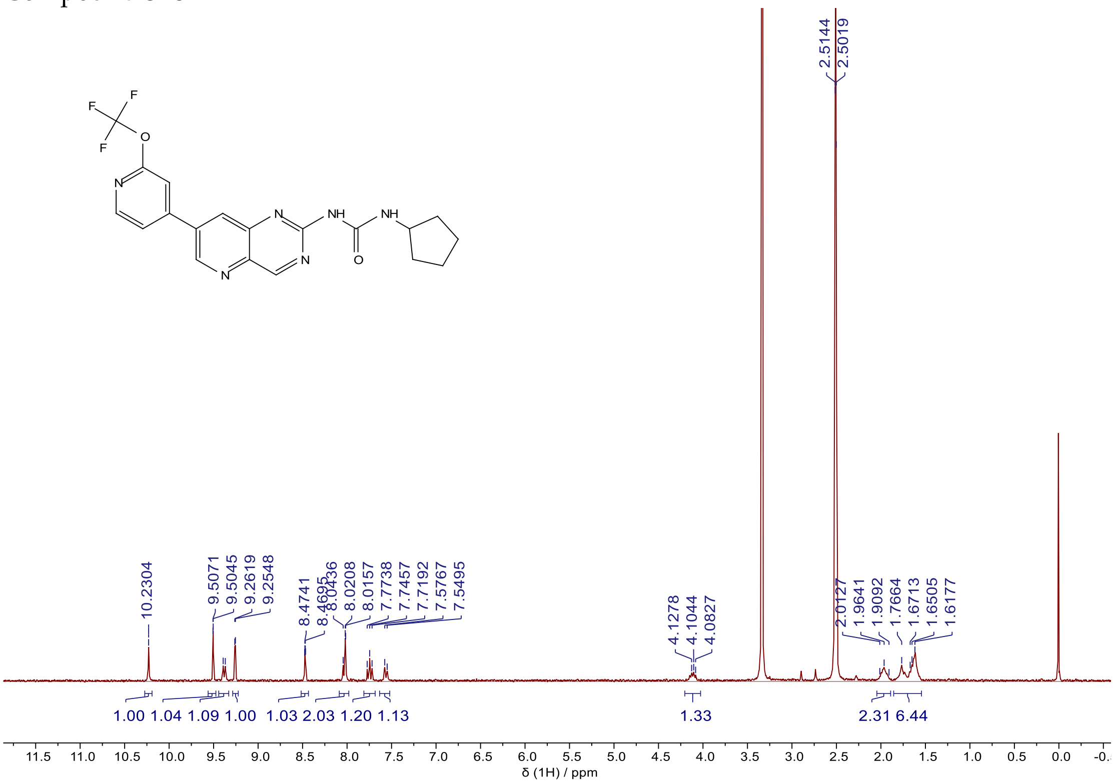

^{1}H-NMR spectra of compound **32c**

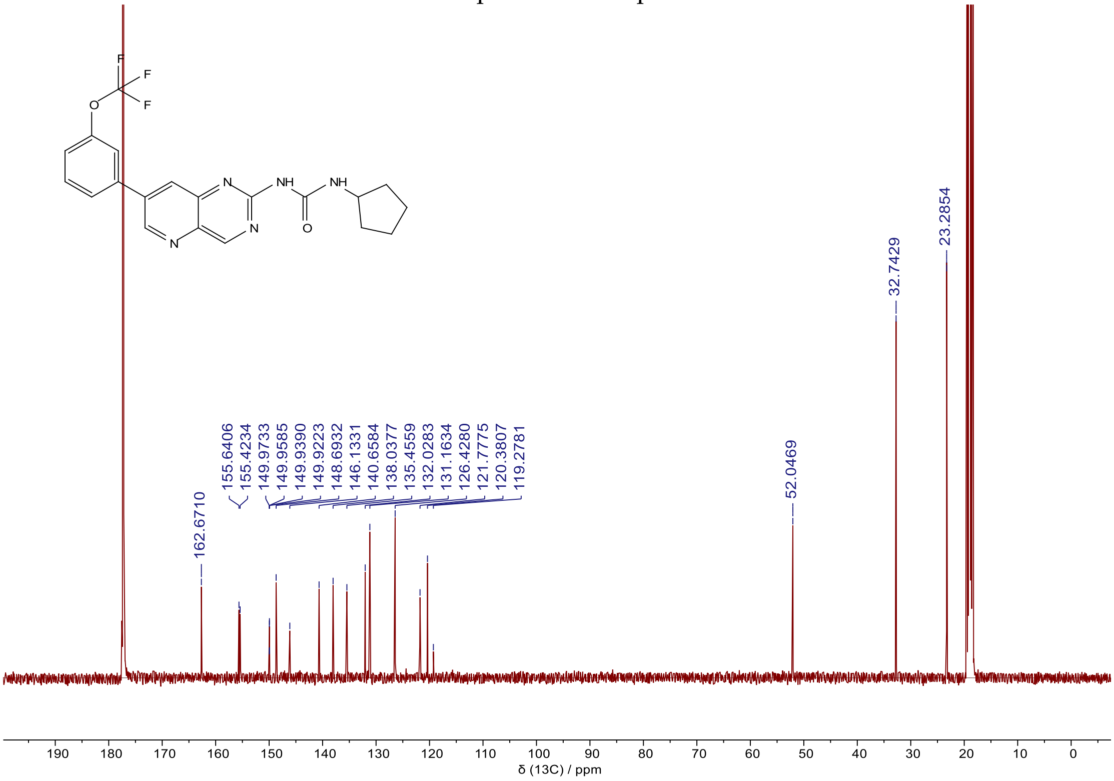

^{13}C-NMR spectra of compound **32c**

Compound **32d**

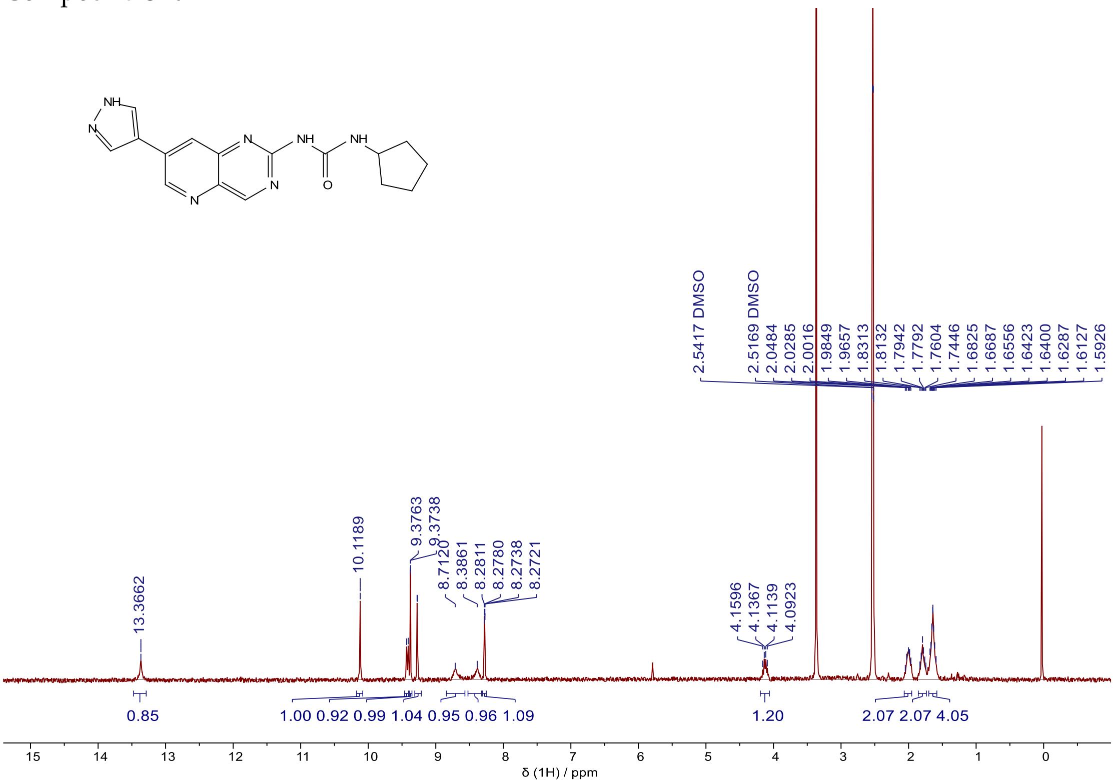

^{1}H-NMR spectra of compound **32d**

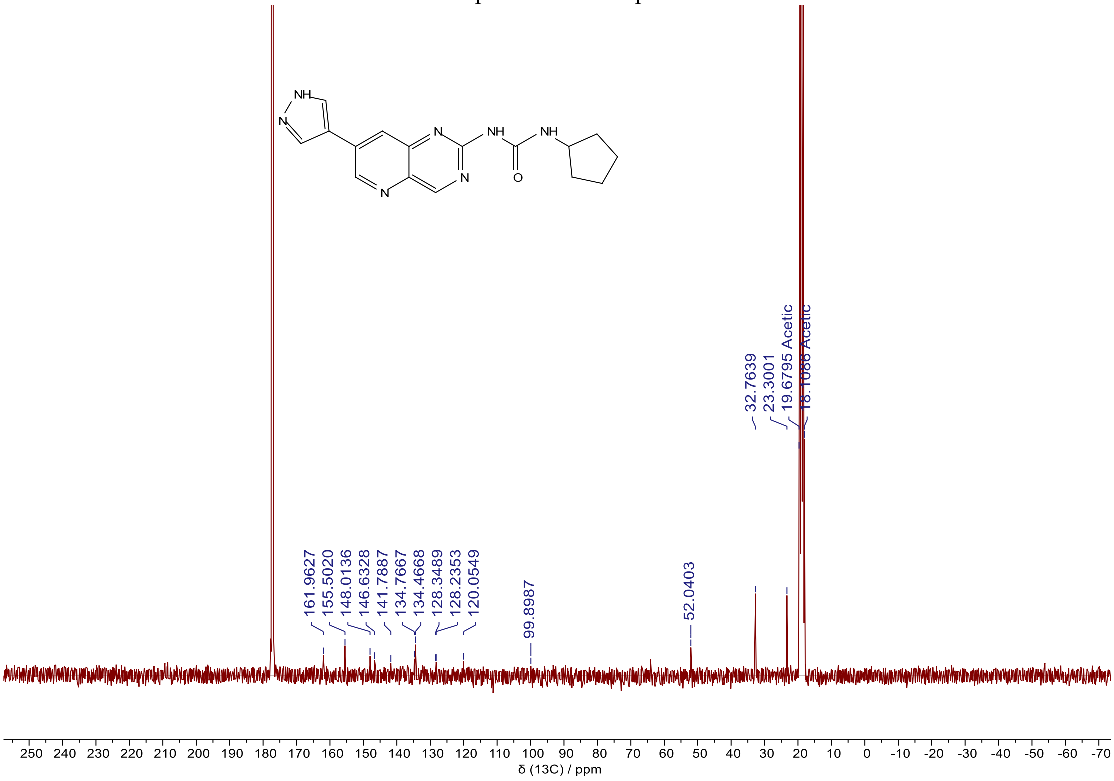

^{13}C-NMR spectra of compound **32d**

Compound **32e**

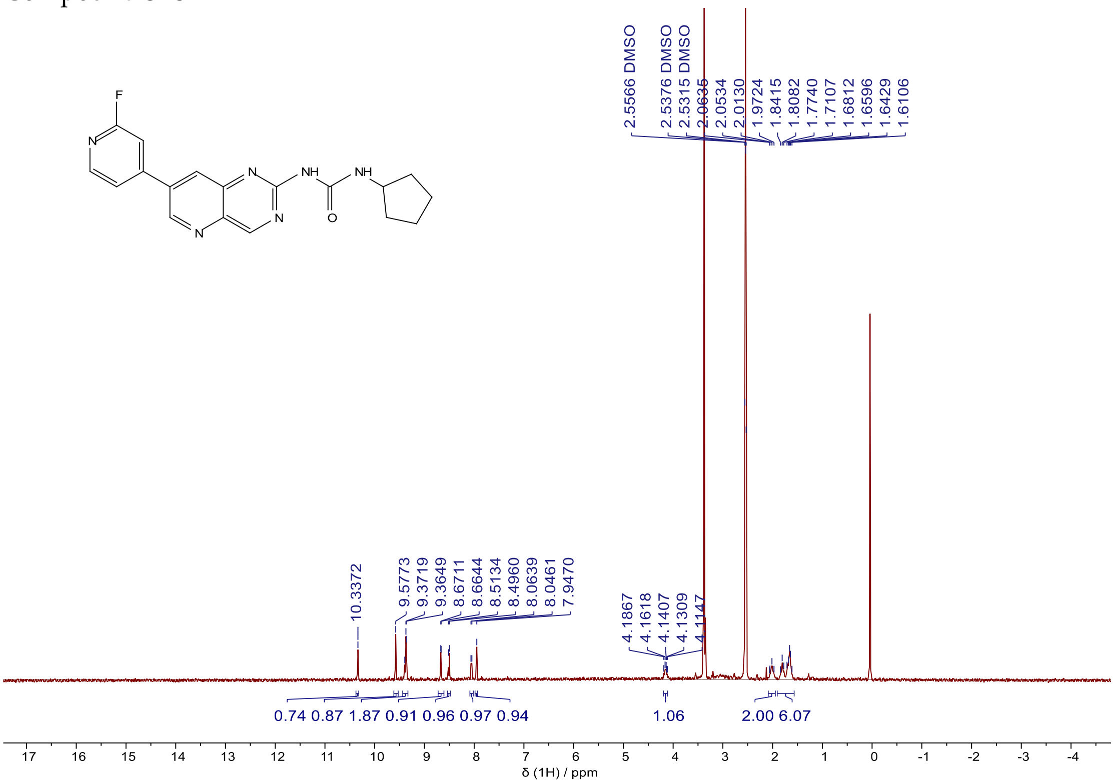

^{1}H-NMR spectra of compound**32e**

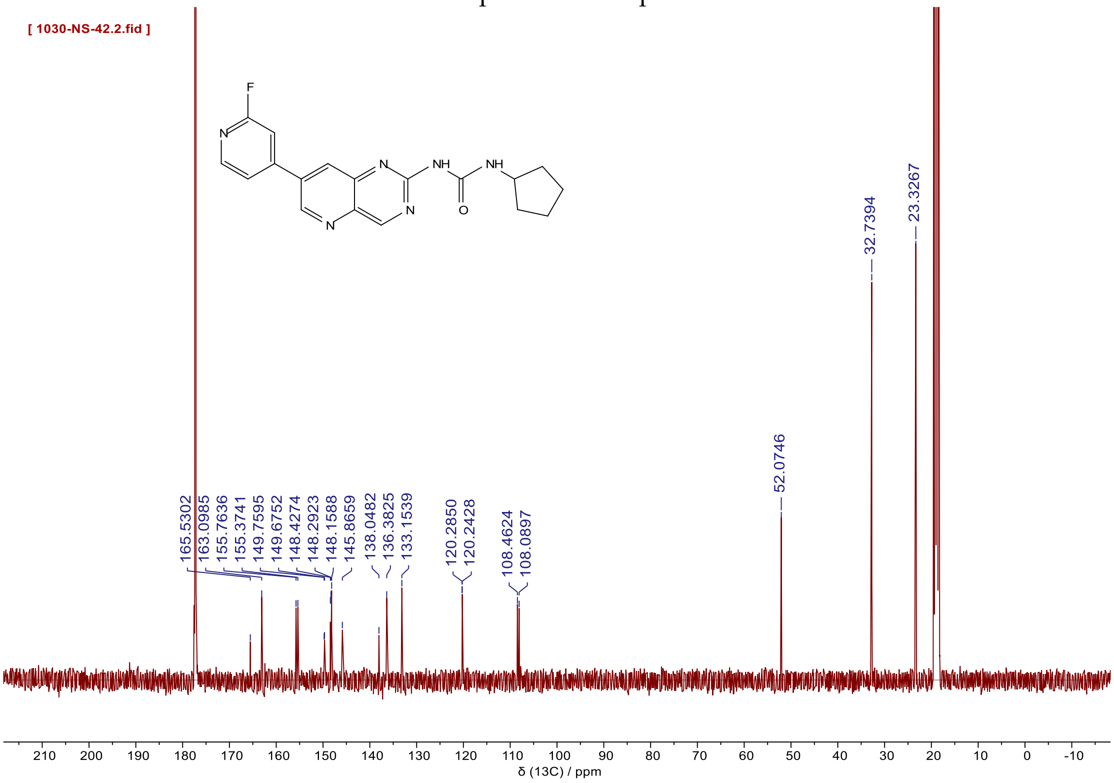

^{13}C-NMR spectra of compound **32e**

Compound **32f**

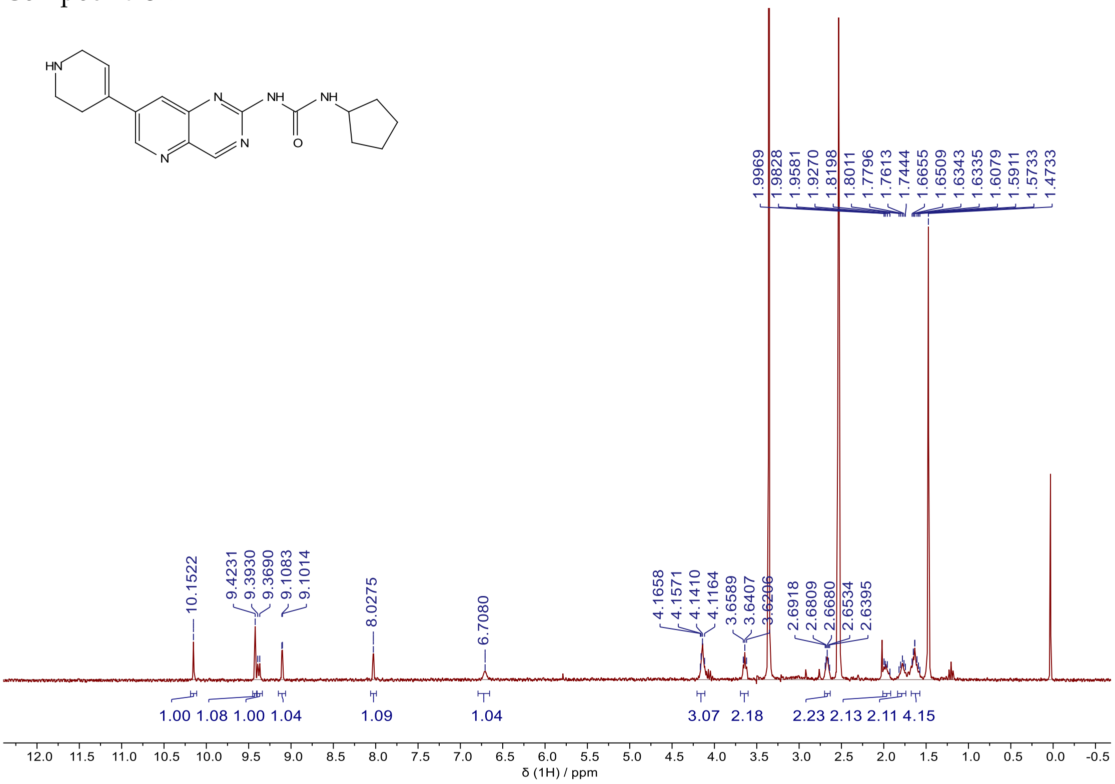

^{1}H-NMR spectra of compound **32f**

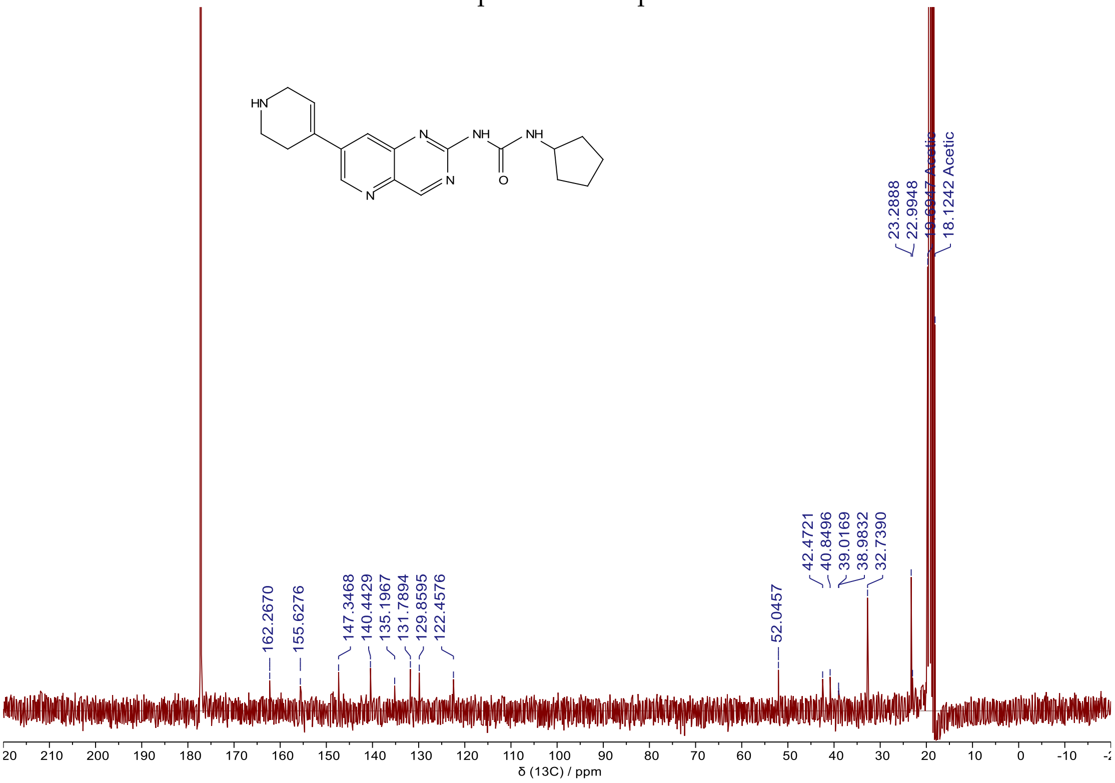

^{13}C-NMR spectra of compound **32f**

Compound **32g**

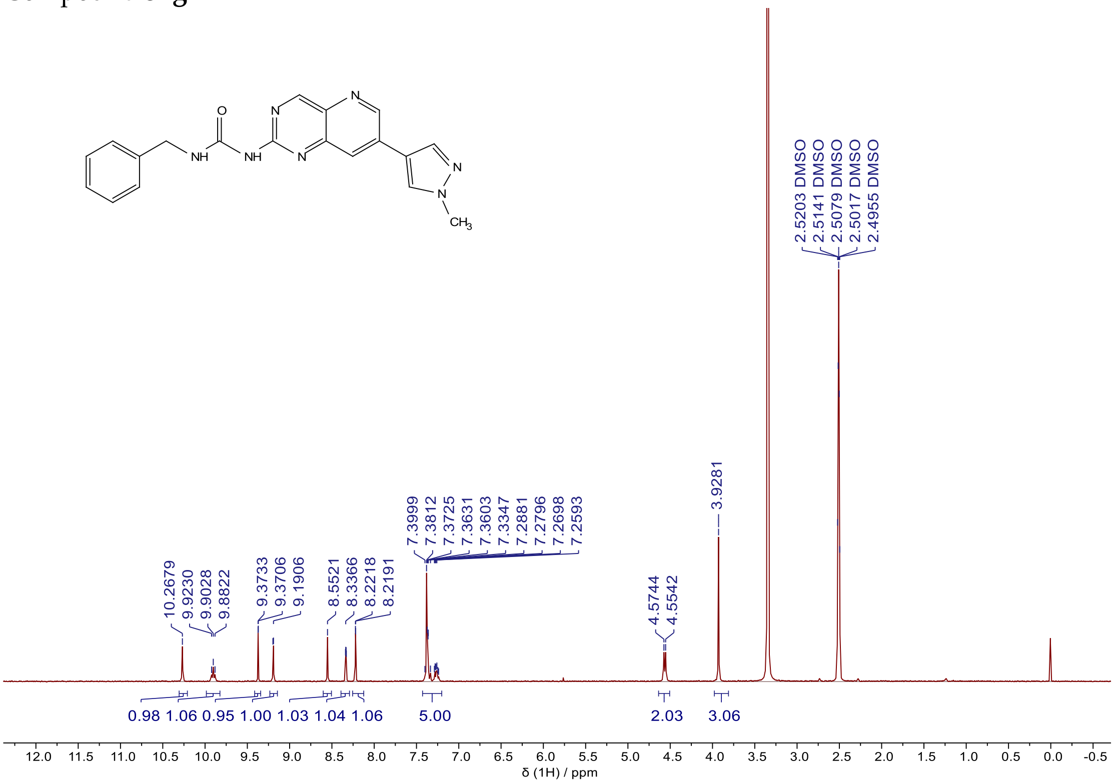

^{1}H-NMR spectra of compound **32g**

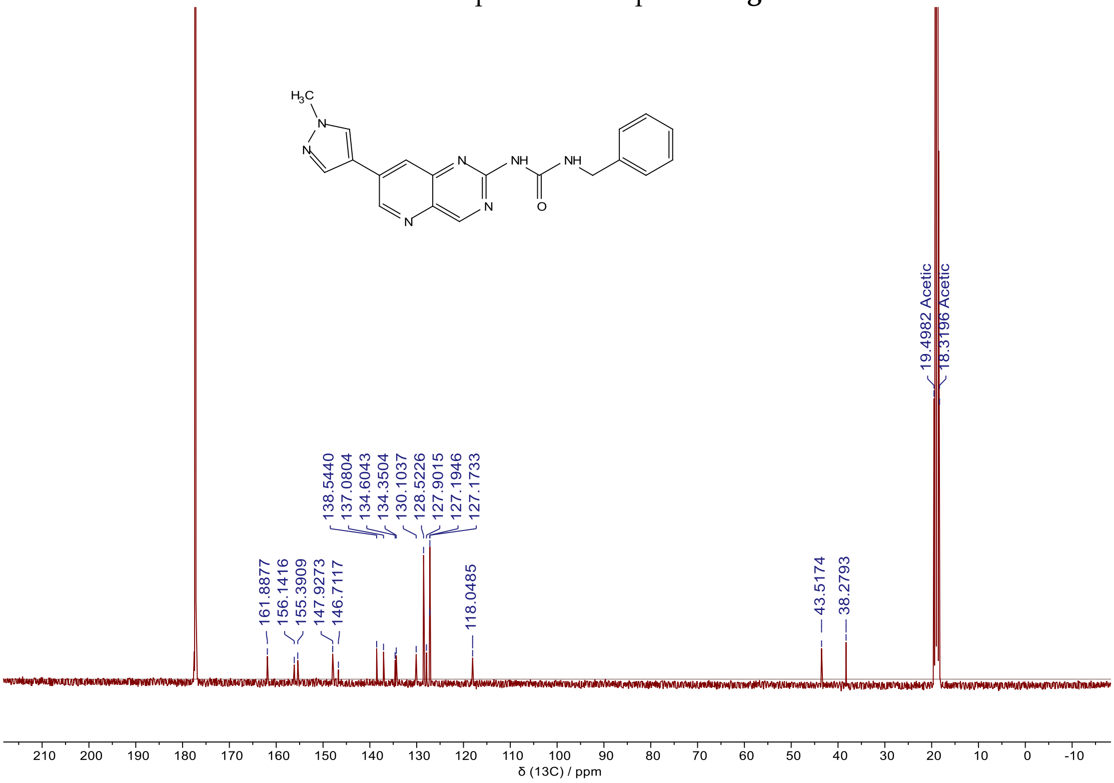

^{13}C-NMR spectra of compound **32g**

Compound **32h**

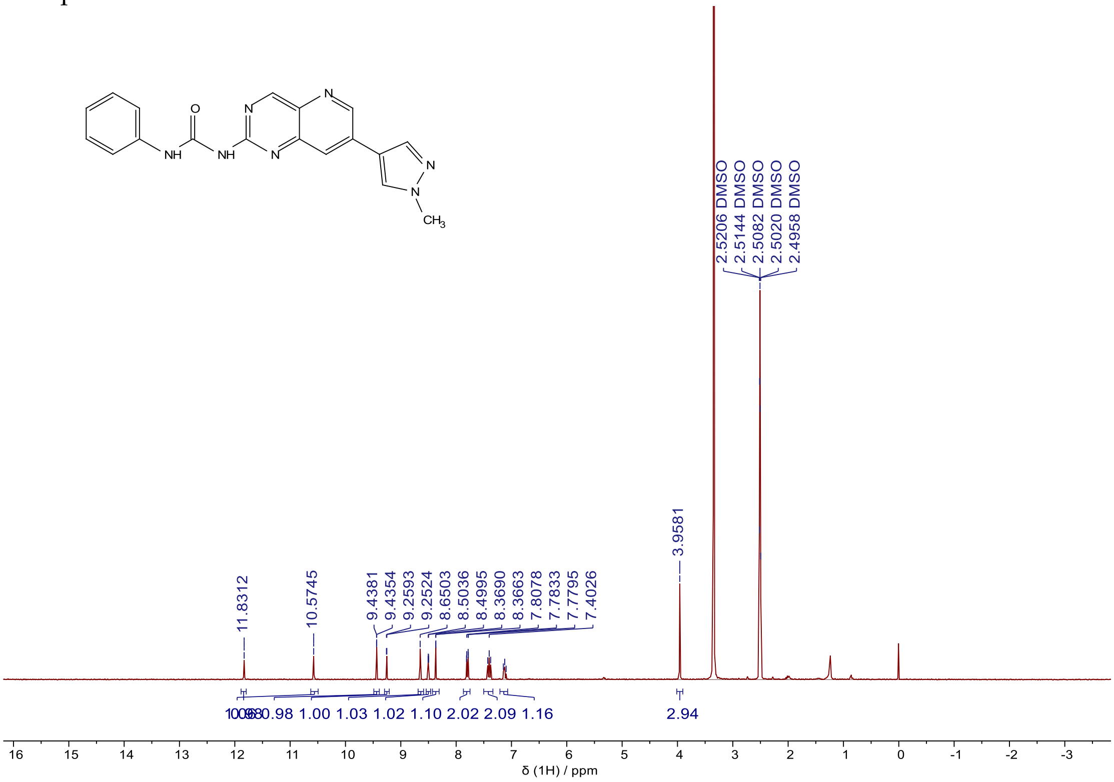

^{1}H-NMR spectra of compound **32h**

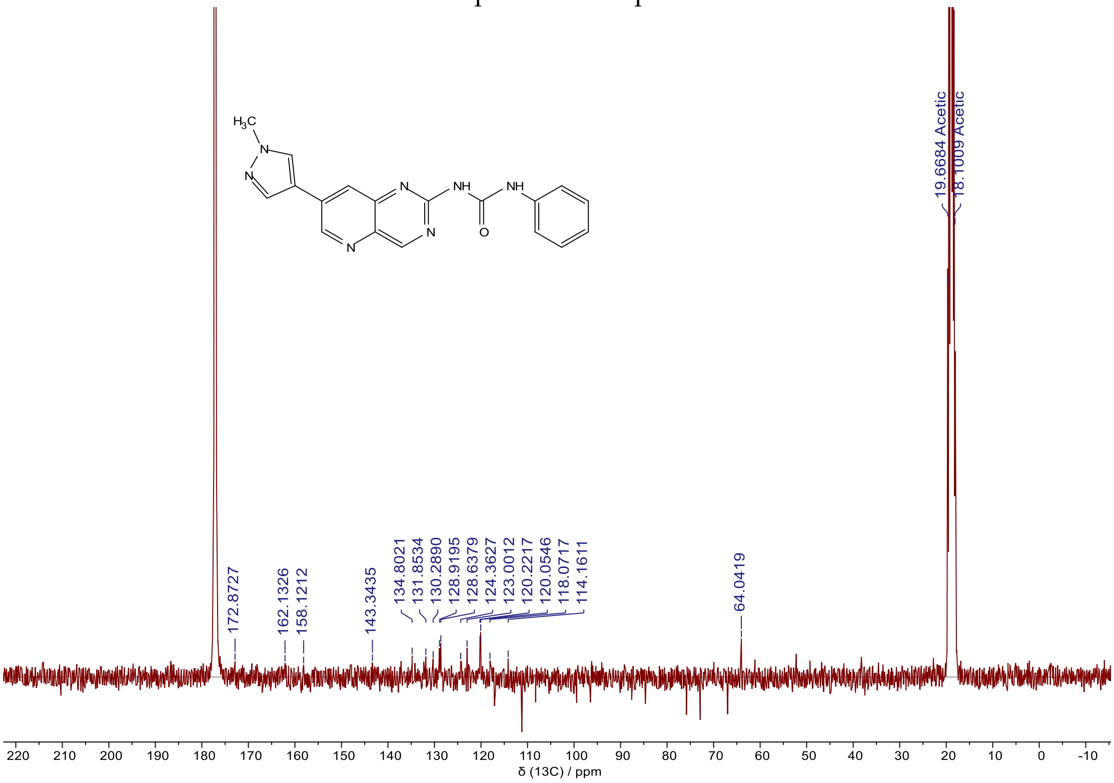

^{13}C-NMR spectra of compound **32h**

Compound **32i**

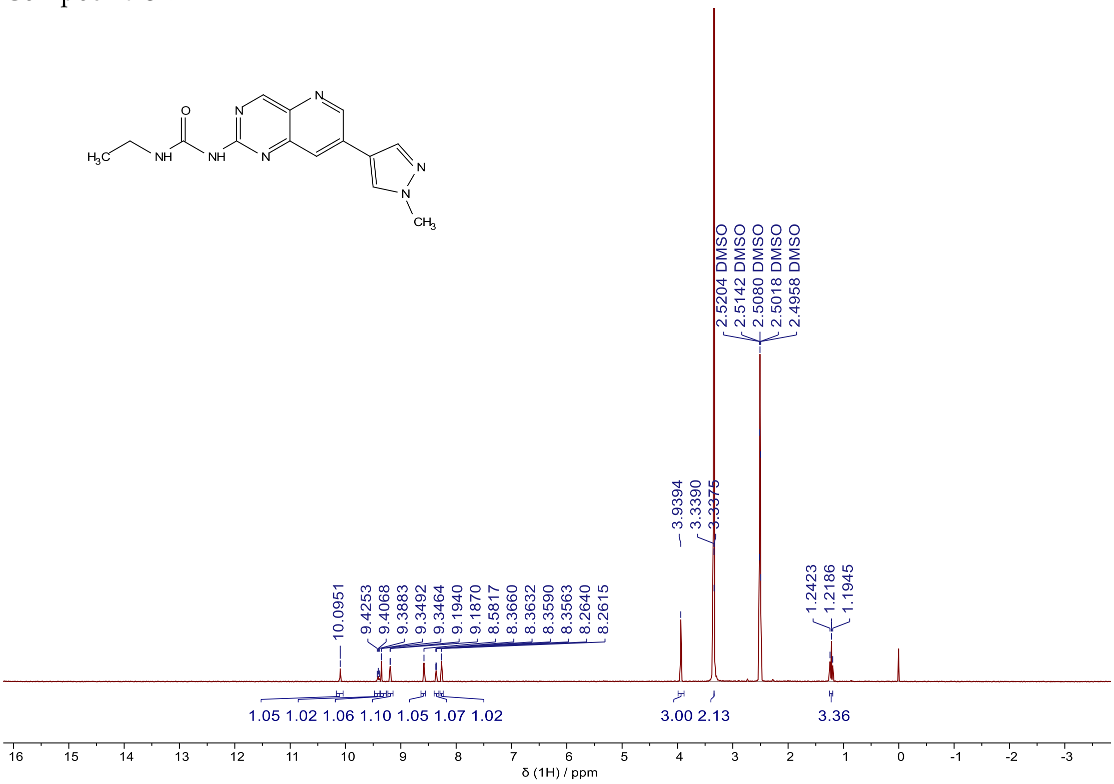

^{1}H-NMR spectra of compound **32i**

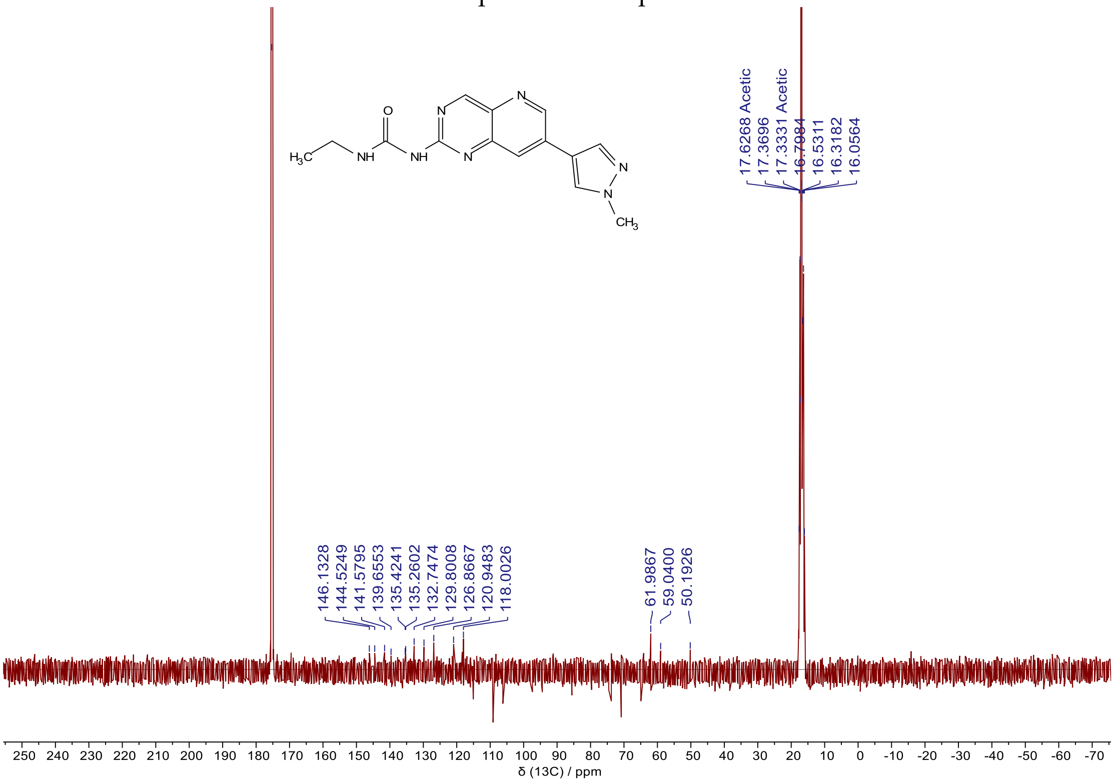

^{13}C-NMR spectra of compound **32i**

Compound **32j**

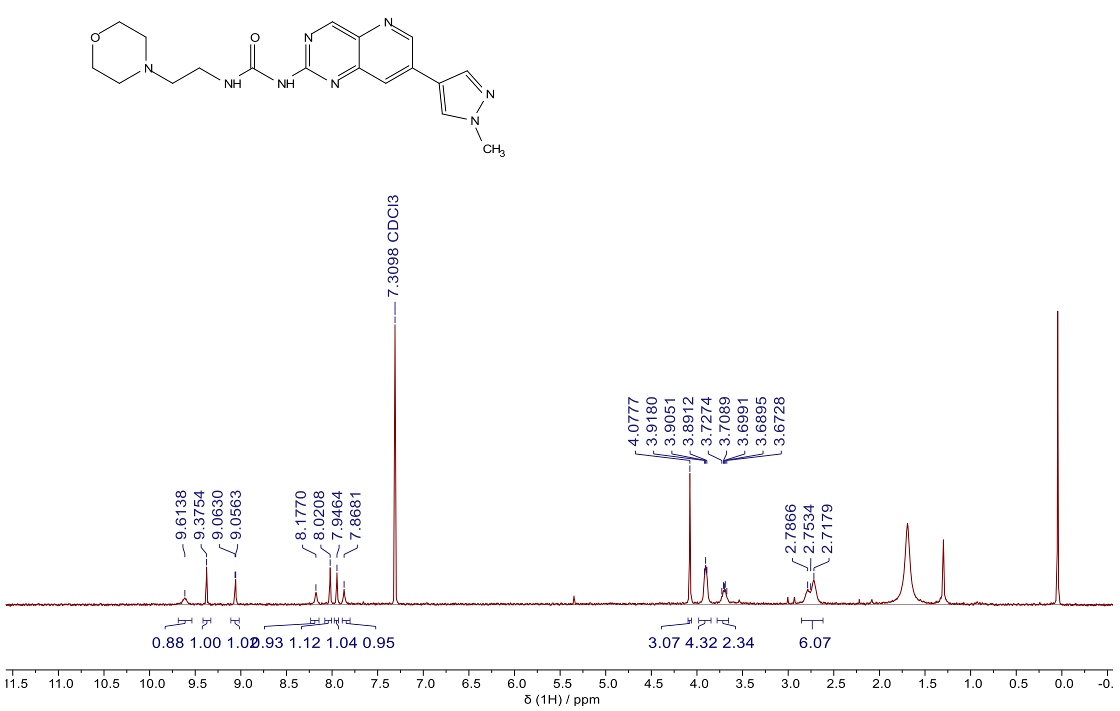

^{1}H-NMR spectra of compound **32j**

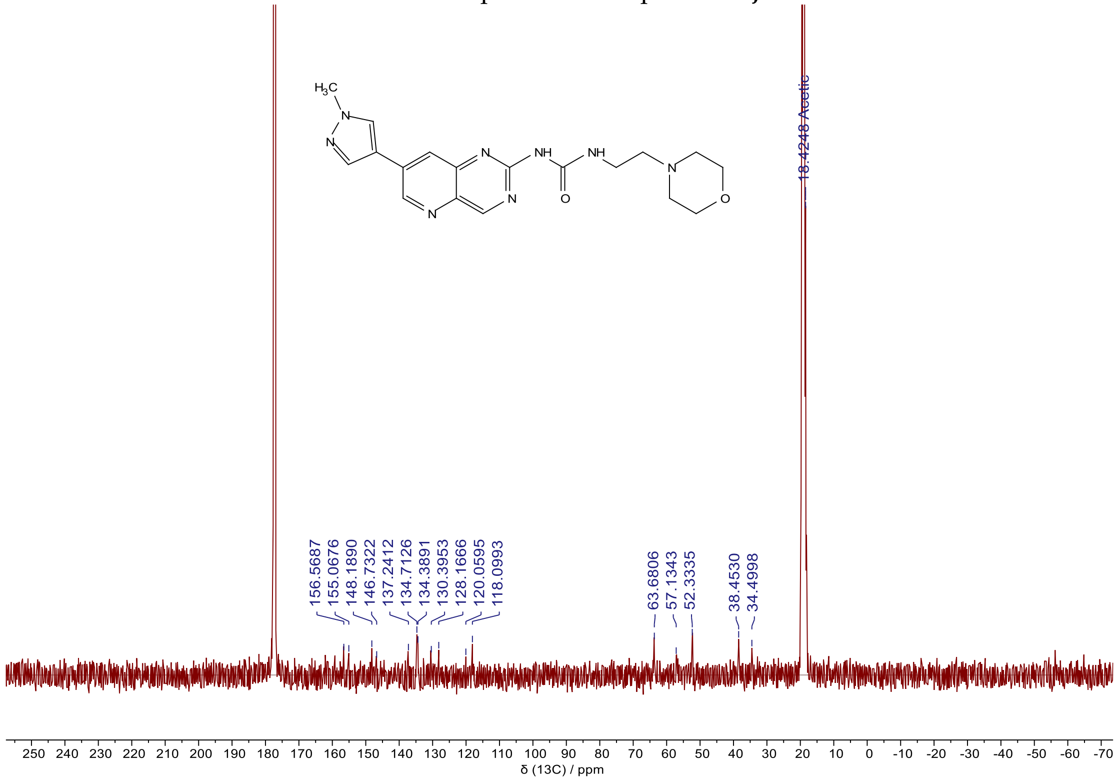

^{13}C-NMR spectra of compound **32j**

Compound **32k**

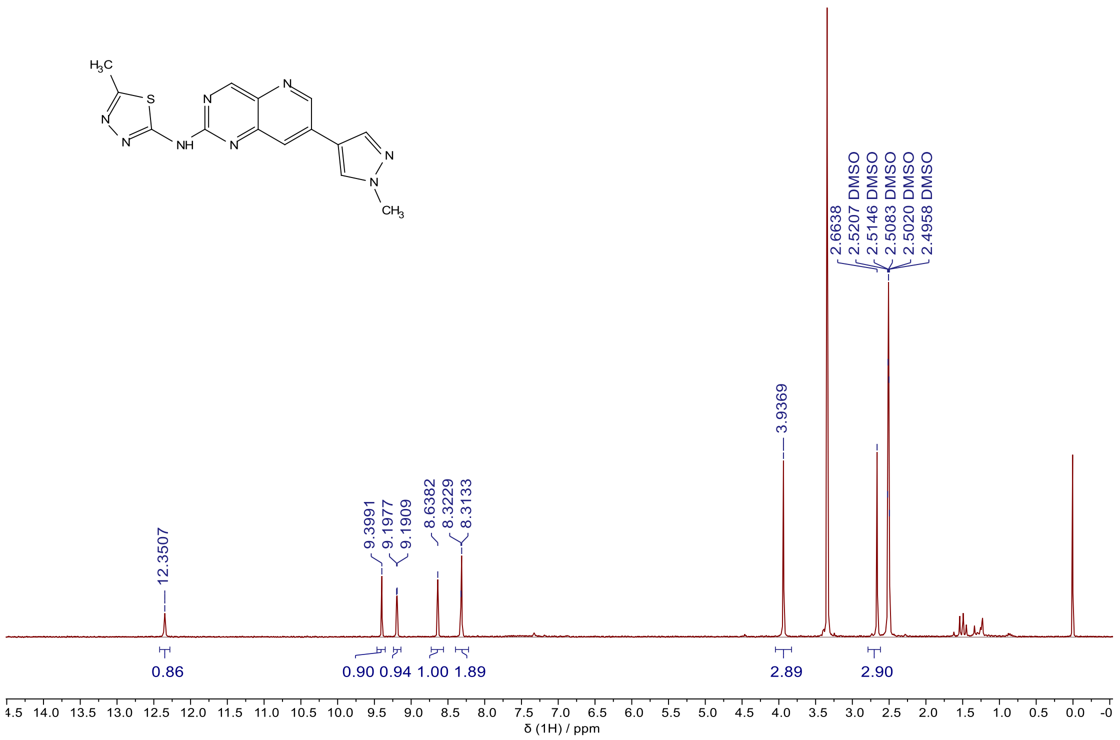

^{1}H-NMR spectra of compound **32k**

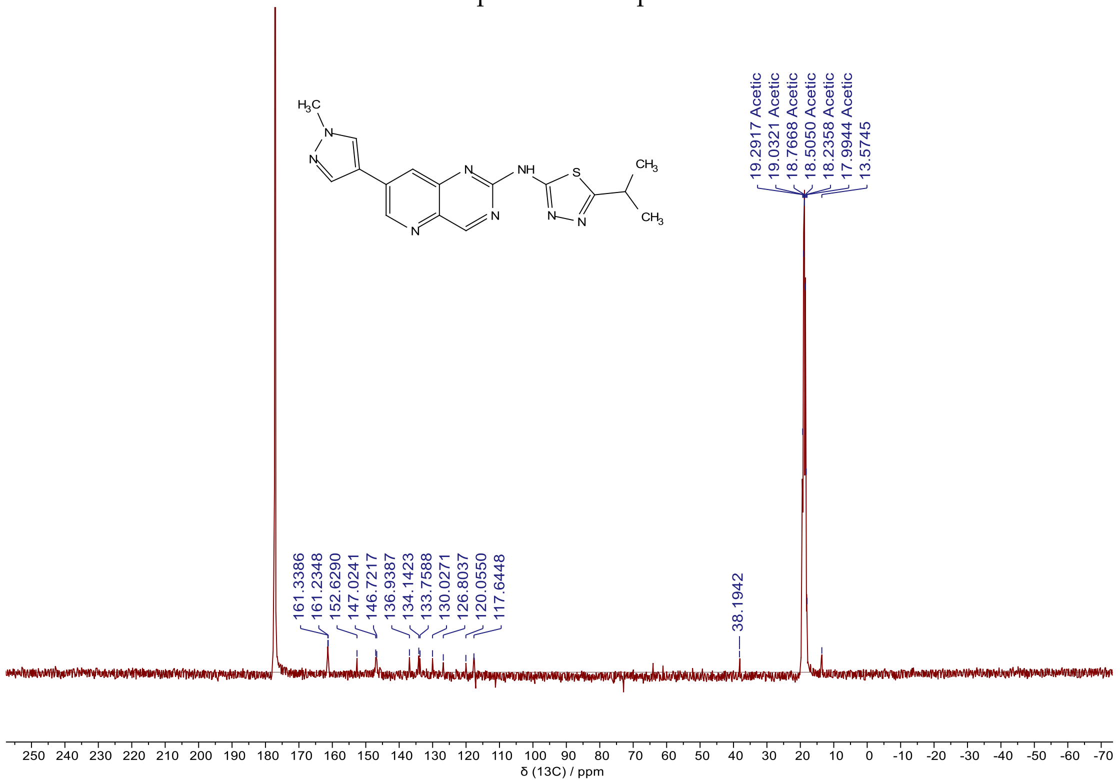

Compound **32l**

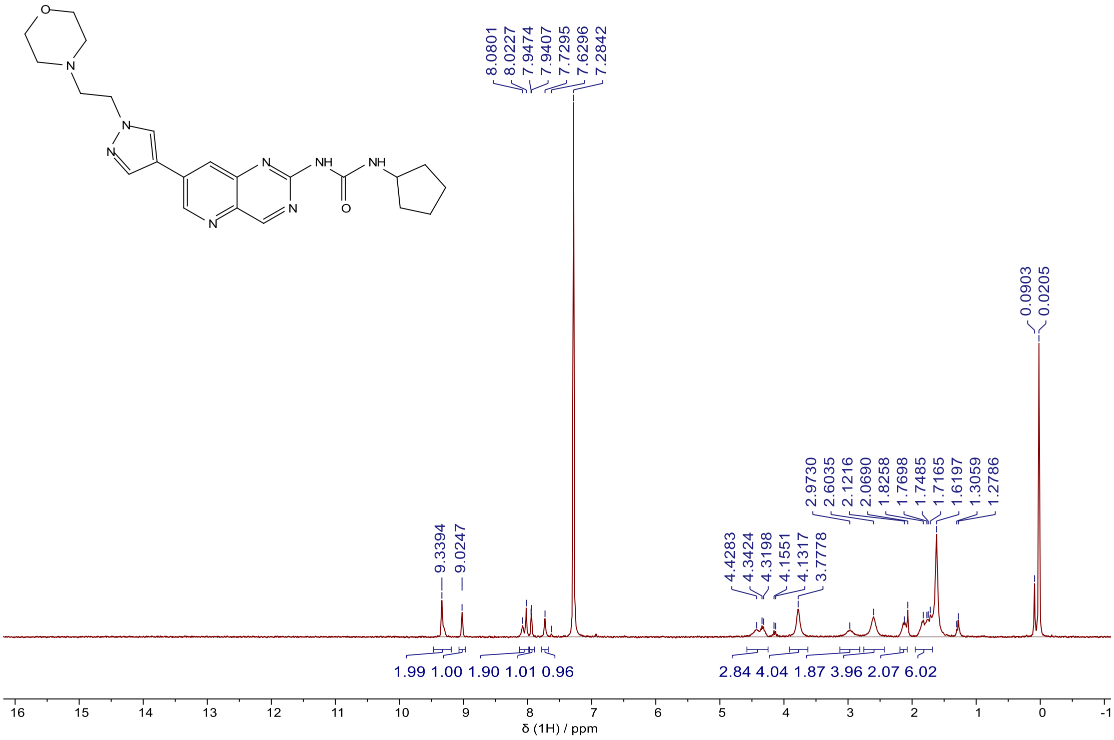

^{1}H-NMR spectra of compound **32l**

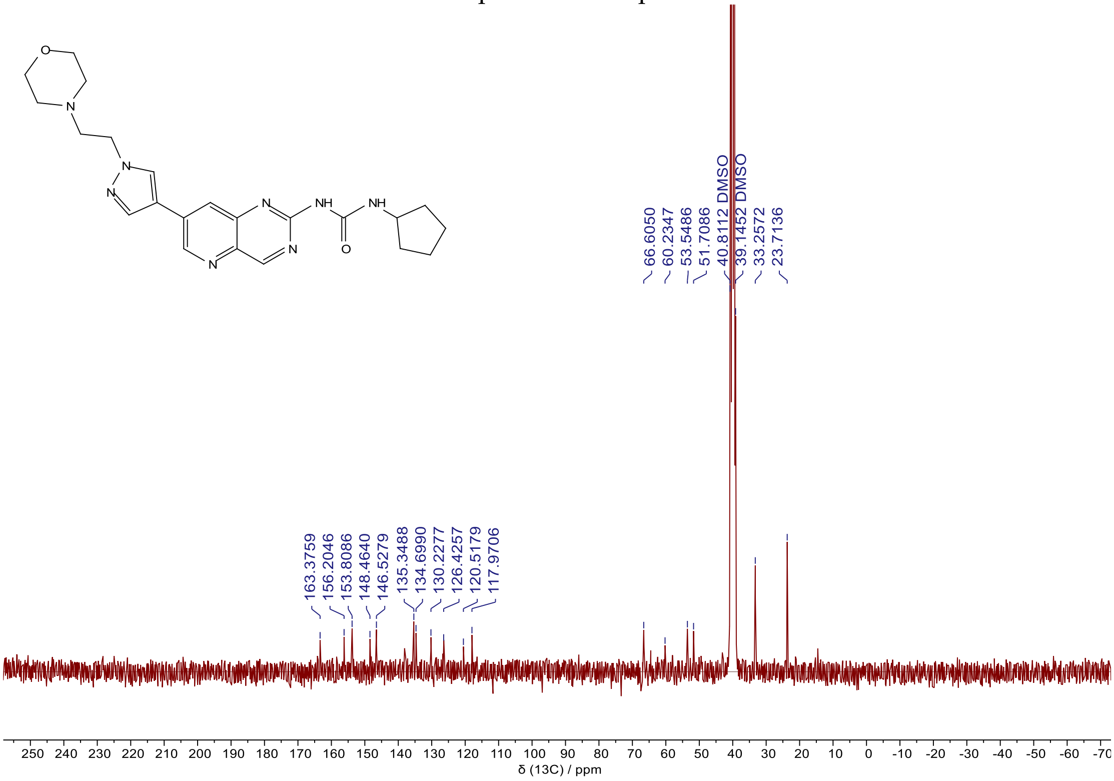

^{13}C-NMR spectra of compound **32l**

Compound **32m**

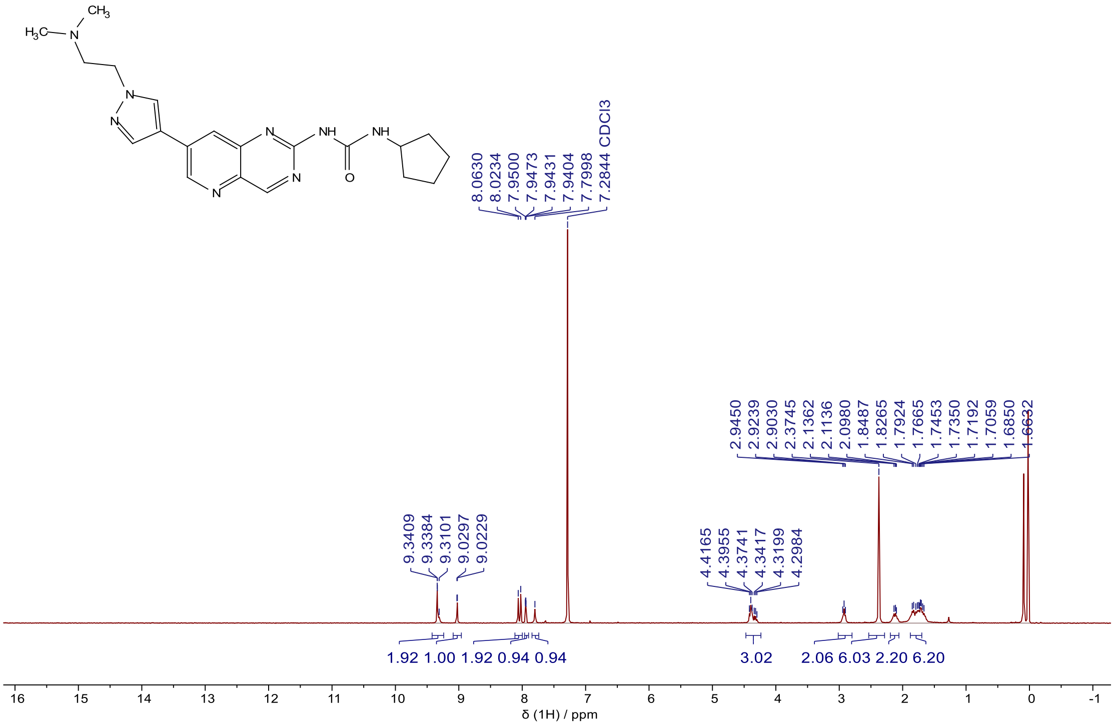

^{1}H-NMR spectra of compound **32m**

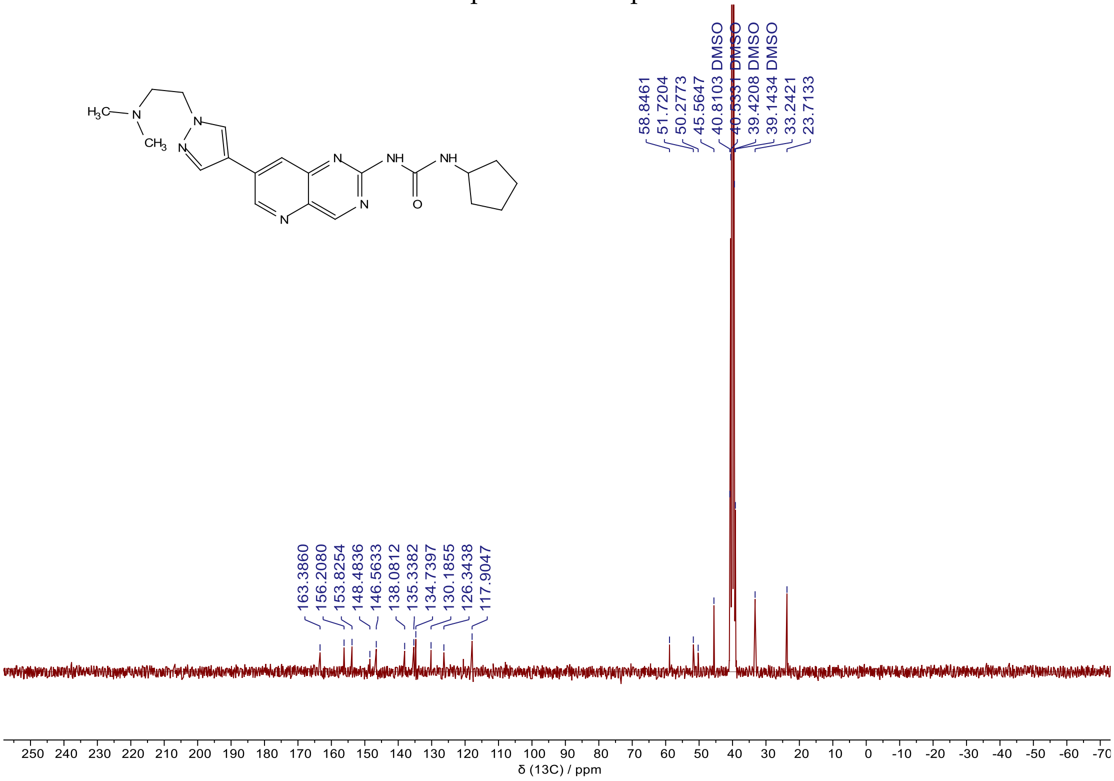

^{13}C-NMR spectra of compound **32m**

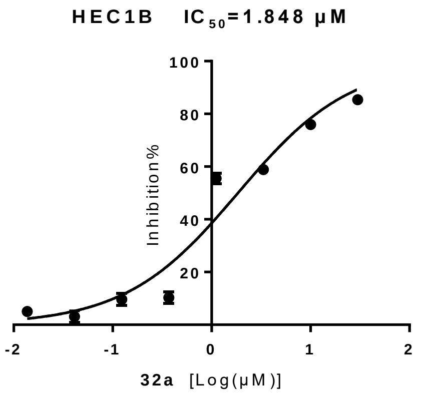
HEC1B IC50=1.848 μM
Inhibition%
32a [Log(μM)]

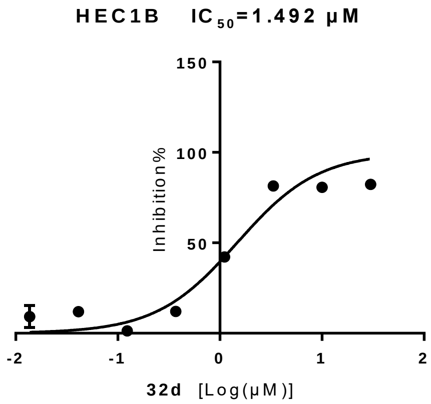
HEC1B IC50=1.492 μM
Inhibition%
32d [Log(μM)]

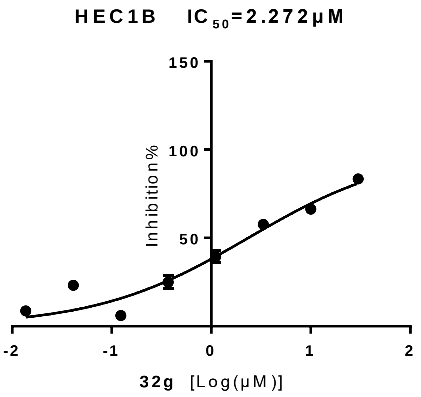
HEC1B IC50=2.272μM
Inhibition%
32g [Log(μM)]

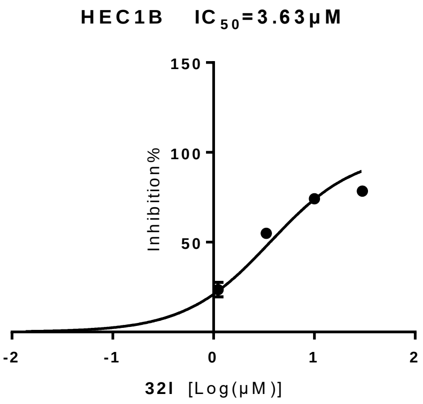
HEC1B IC50=3.63μM
Inhibition%
32l [Log(μM)]

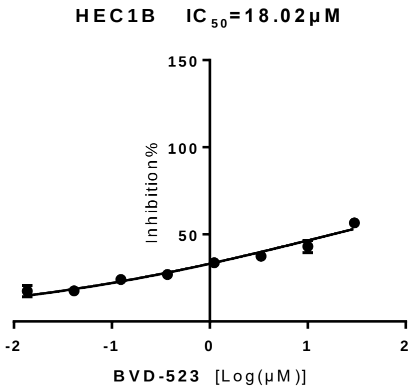
HEC1B IC50=18.02μM
Inhibition%
BVD-523 [Log(μM)]

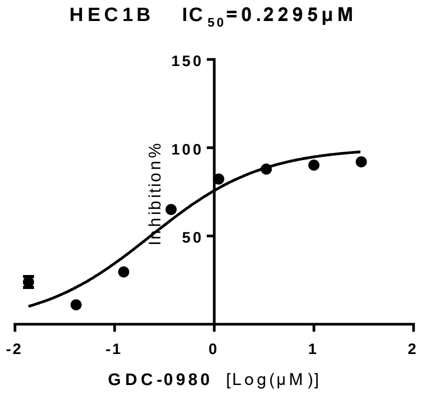
HEC1B IC50=0.2295μM
Inhibition%
GDC-0980 [Log(μM)]

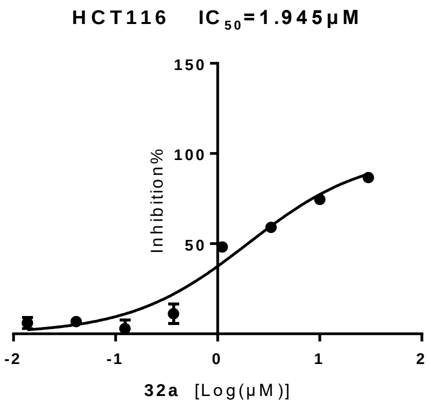
HCT116 IC50=1.945μM
Inhibition%
32a [Log(μM)]

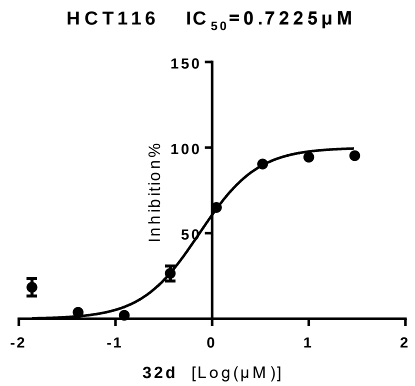
HCT116 IC50=0.7225μM
Inhibition%
32d [Log(μM)]

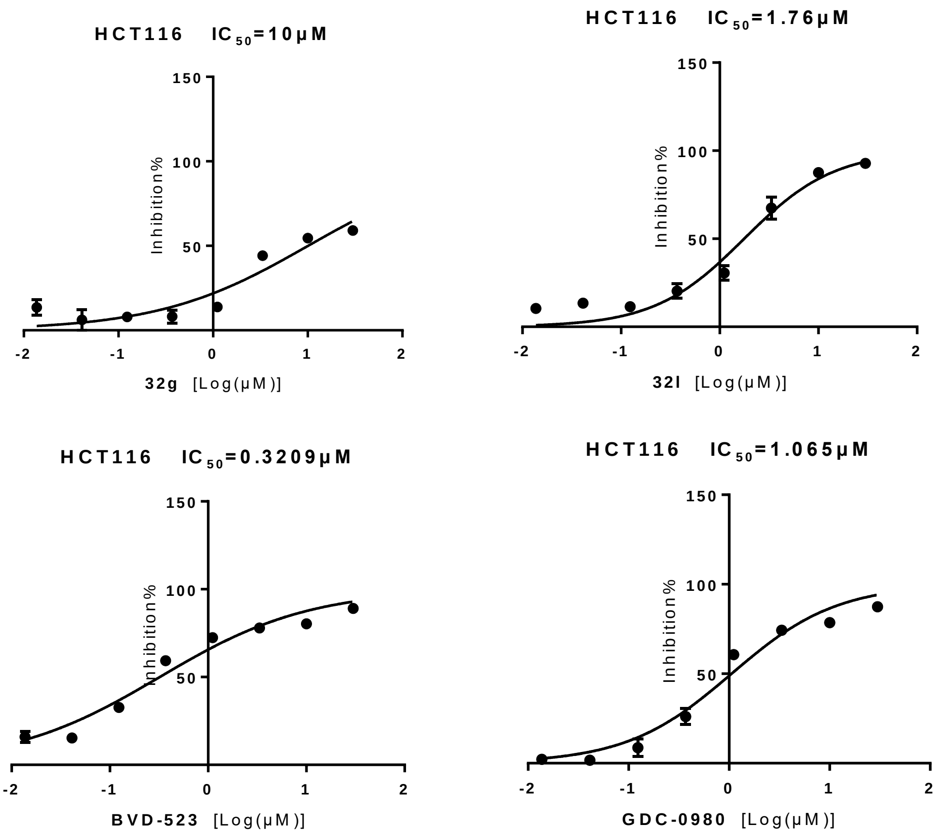

Figure S1. Dose-inhibition response curves of compounds **32a**, **32d**, **32g**, **32l**, BVD-523 and GDC-0980.
